# A unique fungal strain collection from Vietnam characterized for high performance degraders of bioecological important biopolymers and lipids

**DOI:** 10.1371/journal.pone.0202695

**Published:** 2018-08-30

**Authors:** Sophie C. Brandt, Bernhard Ellinger, Thuat van Nguyen, Quyen Dinh Thi, Giang van Nguyen, Christiane Baschien, Andrey Yurkov, Richard L. Hahnke, Wilhelm Schäfer, Martin Gand

**Affiliations:** 1 Department of Molecular Phytopathology, University Hamburg, Hamburg, Germany; 2 Department ScreeningPort, Fraunhofer Institute for Molecular Biology and Applied Ecology IME, Hamburg, Germany; 3 Institue of Biotechnology, Vietnam Academy of Science and Technology, Cau Giay, Hanoi, Vietnam; 4 Faculty of Biotechnology, Vietnam National University of Agriculture, Trâu Quỳ, Gia Lâm, Hanoi, Vietnam; 5 Leibniz Institute DSMZ—German Collection of Microorganisms and Cell Cultures, Braunschweig, Germany; Kansas State University, UNITED STATES

## Abstract

Fungal strains are abundantly used throughout all areas of biotechnology and many of them are adapted to degrade complex biopolymers like chitin or lignocellulose. We therefore assembled a collection of 295 fungi from nine different habitats in Vietnam, known for its rich biodiversity, and investigated their cellulase, chitinase, xylanase and lipase activity. The collection consists of 70 isolates from wood, 55 from soil, 44 from rice straw, 3 found on fruits, 24 from oil environments (butchery), 12 from hot springs, 47 from insects as well as 27 from shrimp shells and 13 strains from crab shells. These strains were cultivated and selected by growth differences to enrich phenotypes, resulting in 211 visually different fungi. DNA isolation of 183 isolates and phylogenetic analysis was performed and 164 species were identified. All were subjected to enzyme activity assays, yielding high activities for every investigated enzyme set. In general, enzyme activity corresponded with the environment of which the strain was isolated from. Therefore, highest cellulase activity strains were isolated from wood substrates, rice straw and soil and similar substrate effects were observed for chitinase and lipase activity. Xylanase activity was similarly distributed as cellulase activity, but substantial activity was also found from fungi isolated from insects and shrimp shells. Seven strains displayed significant activities against three of the four tested substrates, while three degraded all four investigated carbon sources. The collection will be an important source for further studies. Therefore representative strains were made available to the scientific community and deposited in the public collection of the Leibniz-Institute DSMZ–German Collection of Microorganisms and Cell Cultures, Braunschweig.

## Introduction

The fungal kingdom contains a huge biodiversity with different morphologies and habitats, ranging from unicellular aquatic chytrids to large mushrooms and even some of the biggest organisms in the world [1]. There are more than 5 million species estimated from which about 5% are formally classified [2,3]. The occurrence of fungi is ubiquitous and they have been found in different environments, among them soil, freshwater and sea water, in plants and animals, either as saprophytes, symbionts or pathogens. This highlights the huge impact of fungi on environment and humanity. A number of human and animal pathogenic fungi cause serious health risks, either by direct infection, like different *Candida* species [4] and *Aspergillus fumigatus* [5] or by poisoning food and feed with mycotoxins like aflatoxins B1, B2, G1 and G2, produced by *Aspergillus flavus* [6]. Plant pathogenic fungi are a constant worldwide threat to a stable and sustainable food production and food security [7,8]. In contrast to the negative impact, fungi and yeast are also used as cell factories in white and red biotechnology [9,10]. The broad ranges of ecological niches occupied by fungi, including extreme habitats with unusual high salt concentrations, extreme pH or temperature [11], resulted in a high metabolic diversity and their ability to efficiently recycle nutrients or degrade waste products. Consequently, fungal strains were optimized to produce different kinds of molecules like organic acids [12], proteins [13], enzymes [14] and small molecule drugs including antibiotics [15]. As producers and refiners, they play a central role for many biotechnological applications in the food and feed [16], pharma [17], detergent [18] and bio-fuel industry [19]. Best described are several species of *Aspergilli*, like *A*. *niger*, the industrial producer of citric acid [20] and other acids, as well as *Trichoderma reesei* as an example for important industrial enzyme production like cellulases and xylanases [21].

In the case of thermostable lipases, which are needed in many processes, the CAL-A and CAL-B lipase (*Moesziomyces antarcticus* (formerly *Candida antarctica)* lipase A and B) are a very successful example. CAL-A is able to operate at temperatures above 90°C [22] and CAL-B is used for kinetic resolution of amines in multiple 100 tons per year scale [23]. Lipases are just one example of a sought after enzyme class. Lignocellulose and chitin degrading enzymes are at least as important. Chitin, the second most abundant biomass on earth [24], could play a substantial role for the production of sugar based products.

Two major enzyme classes involved in the degradation of the lignocellulose complex are cellulases and xylanases. They are used for the fermentation of plant biomass towards bioethanol. Although bioethanol serves nowadays mainly as energy source it can also serve as the basis for ethylene production, currently performed on a scale of 100 million tons per year [25]. In general, enzymatic degradation enables the direct application of otherwise not valorized by-products like straw or shrimp shells, a natural source of chitin.

In order to find possible new and efficient biocatalysts acting on the aforementioned waste products, it is beneficial to use an unbiased approach and to cover different environments and habitats. Due to the high occurrence of lignocellulose in plant waste-products (up to 40%) as well as the increasing utilization of chitin and lipids, novel enzymes are urgently needed to biotransform these materials into valuable products. There have been many researches in this field of biotransformation, but efficient valorization is still an ongoing task. In our work, we focused on fungi collected in Vietnam, as it belongs to one of twenty-five countries considered to possess a uniquely high level of biodiversity [26]. Vietnam is within the Indo-Burma Biodiversity Hotspot (IBBH), which hosts 110 Key Biodiversity Areas [27]. The fungal diversity of Vietnam still remains vastly unknown, but as 10% of Vietnam’s plants as well as 180 vertebrates are endemic in Vietnam [28], a high diversity is assumed. Vietnam is also among the top 10 of the largest rice and shrimp producers of the world, making it an ideal starting point for bioprospecting to identify new lipases, cellulases, xylanases and chitinases for biotechnological approaches. Within a bilateral research project (BMBF, funding number: 03A0150B) we collected fungi in Vietnam originating from different habitats within Vietnam and characterized their enzymatic profiles.

## Materials and methods

### Collection of fungi in Vietnam

Fungal strains were obtained from a collection in August-September 2014 at nine types of substrates (habitats): decaying rice straw, decaying wood, soil, insect’s bodies (entire dead insects), crab shell (dead), hot springs, shrimp shell (dead), decaying fruits and oil environment (butchery waste). These substrates were found at six environments in north, middle and south of Vietnam (three spots in and around Hanoi, Vinh, Hue and Ho-Chi-Minh City), the geographic origin can be found in S1 Table.

Cultivation experiments were performed in Vietnam. To isolate the strains from the different substrates, 10 g of material were dissolved with sterile water in sterile 50 mL—conical centrifuge tubes, vortexed one minute, followed by ten-fold serial dilutions performed. 100 μL of each dilution was pipetted on PDA plates (Potato Dextrose Agar, order number X931.2, Karl Roth, Germany,) to cultivate the mixture of microbes. After incubation at 28°C for 3 to 5 days, visibly pure cultures were selected and prepared for transport to Germany, including all necessary documents. The collection was permitted by Prof. Dr. Quyen Dinh Thi, while no other requirements for sampling locations were needed. While sampling no endangered or protected species were involved. All isolates were sub-cultured at the University Hamburg, Department of Molecular Phytopathology, on PDA agar plates. The collection was stored as an aqueous conidia suspension at −80°C for each fungus. The conidia were produced by growing mycelia on PDA plates and washed from the surface with ddH_2_O. Representative strains characterized by distinct enzymatic profiles were deposited at the Leibniz-Institute DSMZ–German Collection of Microorganisms and Cell Cultures, Braunschweig (S1 Table).

### DNA isolation and ITS sequencing

The DNA of all fungal strains was isolated with the CTAB method as described previously [29,30]. The internal transcribed spacer regions (ITS-1, 5.8S rRNA, ITS-2) were identified and assigned as described [31–33]. The ITS-1/5.8S rRNA/ITS-2 region was amplified and sequenced with primers ITS1_fw: TCCGTAGGTGAACCTGCGG and ITS4_rv: TCCTCCGCTTATTGATATGC according to White et al. [34]. Sequencing reactions were performed using the ABI Dye Terminator technology according to the manufacturer’s instructions (Applied Biosystems, Foster City, CA, USA). From 183 strains high quality ITS sequences could be amplified. After sequencing and chimera check 164 strains were identified (19 putative chimeras). The phylogenetic tree was inferred from sequences of the internal transcribed spacer 1, the 5.8S rRNA gene and the internal transcribed spacer 2, aligned with poa [35] and gblocks [36]. Phylogenetic tree was inferred under the maximum likelihood (ML) and maximum parsimony (MP) criterion [37]. Reference ITS sequences have been retrieved from MycoBank [38]. The nomenclature of *Trichoderma* strains was verified using *Trich*OKEY 2 [39]. All ITS sequences of this study were deposited in GenBank, numbers are given in S1 Table. The best matching results of the ITS sequence blast with type species and relation of all fungi can be found in the S1 Table.

### Enzymatic analysis

Enzymatic analysis was performed in two steps: the first step was a plate based assay to qualify the enzyme activity for selected enzymes (as described in Plate-based enzyme screening). The second step was the quantitative evaluation of the supernatant of the previously selected fungi (as described in Quantification of enzyme activity of the supernatant).

#### Plate-based enzyme screening

The plate-based screening of actively growing fungi is based on the disc diffusion method for antimicrobial susceptibility testing by Bauer et al [40]. Even though this method was invented to determine antimicrobial susceptibility, in this study we show the wide range of its application. The protocol was modified in essential points: The fungus was pre-cultured in liquid potato-dextrose media (Potato Extract Glucose Broth, order number CP74.2, Karl Roth, Germany) for three days at 28°C and 150 rpm. Mineral salt media [41] were used for three substrates, each with 1%, to induce the corresponding secreted enzyme activity. To determine cellulase activities CMC (carboxymethyl cellulose, order number 419273, Sigma-Aldrich, Germany), for xylanase activities xylan from beechwood (Sigma-Aldrich, X4252, Germany) and for chitinase activities powdered chitin from crab shells, (Sigma-Aldrich, order number C7170 Germany) were used. For lipase activity a modified method according to von Tigerstrom et al. [42] with 1% Bacto^TM^ Peptone (Becton, Dickinson and Company, order number 211820, USA), 0.01% CaCl_2_ (Karl Roth, order number CN93.2, Germany) and 1% Tween 20 (Karl Roth, order number 9127.2, Germany) was used. A hole was punched with a 7 mm cork borer in the middle of the 2% agar (Karl Roth, order number 5210.2, Germany) a petri dish. The agar contained either mineral salts with CMC, xylan or chitin, or Bacto Pepton-Tween 20. 20 μL of the pre-cultured supernatant, from the liquid potato-dextrose media, were inoculated into the hole and incubated at 28°C for three days in the dark. Agar plates for chitinase, cellulase and xylanase were stained with Lugol's iodine [43]. The diameter of the white halo from reduced sugars corresponds to the various enzyme activities. In the presence of lipase activity occurs a precipitation of the calcium fatty acids salts released from Tween 20, which is visible on the agar plate. The halo around the cork borer hole was measured and high activity was assigned beyond 1 cm, low activity was defined as between a visibly detectable zone of around 2 mm and up to 1 cm. Exemplary pictures of the plate-based screening are shown in S2 Fig.

#### Quantification of enzyme activity of the supernatant

Based on the plate screening assay 69 fungal strains showing high activity in the afore described plate-based screening were selected for further detailed analysis and 145 activity assays were performed. Strains were pre-cultivated in Yeast extract peptone dextrose media [44] for 3–5 days, mycelia were washed, dried with filter paper (Whatman^®^) and 0.1 g of semi-dried mycelia were incubated with 50 mL of inductive media, containing 1% of the respective inducer (CMC, xylan, chitin or Tween20). Mycelia were incubated for 3 days at 28°C with 145 rpm on a shaker in the dark followed by centrifugation (4000 rpm, 20 min), the supernatant of these induction cultivation was used for the assays. In the case of xylanase, chitinase and cellulase activity 1.5 mL of induction cultivation supernatant was incubated with 1.5 mL 2% (w/v) substrate (CMC; chitin from shrimp shells, powder; xylan from birch wood, order number 95588; all Sigma-Aldrich, Germany) in 50 mM sodium acetate pH 6.5 in 37°C for 2 h under constant shaking at 300 rpm. Enzyme activity was determined by quantifying the resulting monosaccharides, N-acetylglucosamine and D-glucose, products of the cellulase or chitinase reaction, by Schales Reagent [45] and D-xylose originating from xylanase activity by DNS assay. DNS assay was done as described previously [46]. Briefly, 100 μL of the enzymatic reaction was added to 400 μL ultrapure water and 1.5 mL DNS solution (260.6 mM sodium hydroxide (Sigma-Aldrich, order number S8045, Germany), 177.2 mM potassium sodium tartrate (Sigma-Aldrich, order umber 217255, Germany), 11 mM 3,5-dinitrosalicylic acid (Sigma-Aldrich, order number 128848, Germany), 5.3 mM phenol (Sigma-Aldrich, order number P1037, Germany), 0.9 mM sodium sulfate (Sigma-Aldrich, order number 204447, Germany) in 15 mL reaction vessels followed by heating in a 100°C water bath for 10 min. The reaction was stopped on ice, transferred to 96 well plates and optical density (OD) was determined at 575 nm and correlated to a reference for quantification.

Chitinase and cellulase activity was determined using Schales Reagent 400 μL of the enzymatic reaction were added to 400 μL Schales Reagent (0.5 M sodium carbonate (Sigma-Aldrich, order number S7795, Germany), 1.5 mM potassium ferricyanide (Sigma-Aldrich, order number 244023, Germany)), incubated for 15 min in a 100°C water bath, stopped on ice and measured at 420 nm similar to DNS assay. Lipase activity was determined in 96 well plates by adding 100 μμL supernatant to 100 μL detection buffer (500 μM p-NPP (Sigma-Aldrich, order number N2752, Germany) in 50 mM TRIS-HCl (VWR Chemicals, order number 85827.297, Germany) pH 7.5). Plates were sealed and incubated under constant shaking at 200 rpm for 2 h prior OD determination at 410 nm [47].

Protein concentration was determined using BCA protein detection kit (Thermo Fischer Scientific, order number 23225, USA) by adding 25 μL supernatant to 200 μL detection solution following manufacturers’ protocol. The specific enzyme activities are given in S2 Table.

## Results

### Characterization of the strain collection

**Fig 1:**
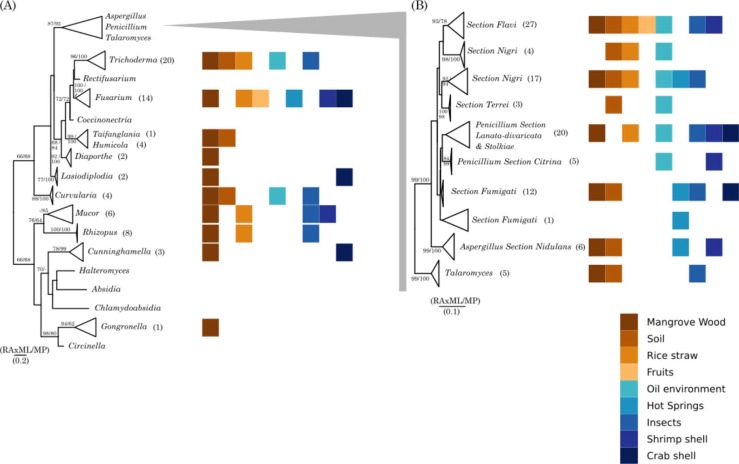
Phylogenetic tree highlighting the position of fungal isolates and closely related type strains. The tree comprising (a) *Ascomycota* and *Zygomycota* strains was inferred from 398 aligned characters and (b) *Aspergillus* and *Penicillium* strains rooted with *Talaromyces* strains was inferred from 393 aligned characters of the internal transcribed spacer 1, the 5.8S rRNA gene and the internal transcribed spacer 2, under the maximum likelihood (ML) and maximum parsimony (MP) criterion. The branches are scaled in terms of expected number of substitutions per site. Numbers adjacent to the branches are support values from 1,000 ML bootstrap replicates (left) and from 1,000 MP bootstrap replicates (right) if larger than 60%. Numbers in parentheses represent the number of strains in this taxon *, nomenclature of *Trichoderma* strains was verified by using *Trich*OKEY 2. The uncollapsed trees can be found in the S3 and S4 Figs. Habitats are wood (dark brown), soil (light brown), rice straw (dark orange), fruits (light orange), oil environment (light green-blue), hot springs (light blue), insects (blue), shrimp shells (dark blue) and crab shells (black-blue).

With the focus on the taxonomic and enzymatic diversity, we used a reductionist approach. In the beginning, fungal strains were collected at different locations and environments (295 strains) in Vietnam, followed by selection due to optical growth differences (211 strains) and DNA isolation (183 strains) and PCR- based comparisons of the ITS sequences (164 strains, ITS1-5.8S rRNA-ITS2). The collection of 295 fungal strains comprised strains mainly from wood, soil, insects, rice straw, shrimp shells, but also from oil environment, crab shells, hot springs and fruits (Table 1). Identified with ITS sequencing, 164 strains were affiliated with 14 different genera, five *Aspergillus* sections and two *Penicillium* sections. The collapsed phylogenetic tree including the original habitats can be found in Fig 1, while the map of Vietnam with sample locations can be found in S5 Fig.

**Table 1:** Overview of the collected fungi and reduced sample amount according to the workflow. The fungal strains were grouped according to their habitats: wood (FW), soil (SF), rice straw (FR), fruits (FF), oil environment (FL), hot springs (FH), insects (Fi), shrimp shell (Fsh) and crab shell (FC). The reductionist approach is described in the DNA isolation and ITS sequencing subsection of the Materials and Methods section.

Habitats	Collected	Optical differences	DNA Isolation and enzymatic activity test (Plate-based)	Enzymatic activity test (supernatant)
**Wood (FW)**	70	38	36	17
**Soil (SF)**	55	42	29	6
**Rice straw (FR)**	44	26	19	3
**Fruits (FF)**	3	2	2	2
**Oil environment (FL)**	24	18	17	7
**Hot springs (FH)**	12	9	5	4
**Insects (Fi)**	47	38	31	18
**Shrimp shell (Fsh)**	27	25	16	11
**Crab shell (FC)**	13	11	9	1
**Ʃ**	295	211	164	69

**Fig 2:**
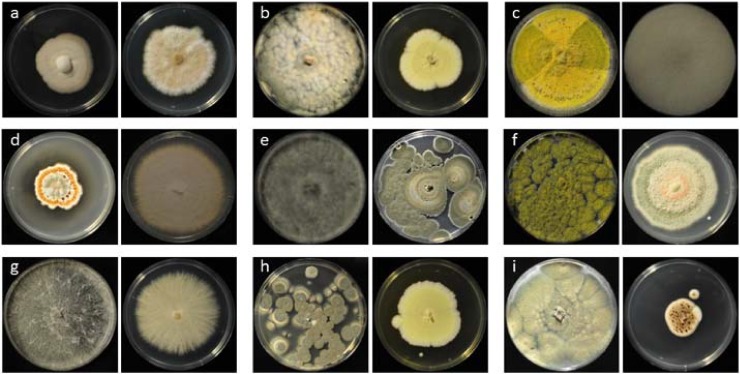
Examples of the collection. a: Fungi from wood (FW57 *Humicola* sp, FW16.1 *Fusarium* sp.), b: Fungi from soil (SF25 *Talaromyces* sp., SF31 *Aspergillus* section *Terrei*), c: Fungi from rice straw (FR1 *Aspergillus* section *Flavi*, FR27 *Trichoderma* sp.), d: Fungi from shrimp shell (Fsh102 *Aspergillus* section *Nidulans*, Fsh13 *Fusarium* sp.), e: Fungi from crab shell (FC2 *Lasiodiplodia* sp., FC7 *Penicillium* section *Lanata-divaricata* & *Stolkiae*), f: Fungi from insects (Fi19 Aspergillus section Flavi, Fi5 *Talaromyces* sp.), g: Fungi from fruits (FF1 *Aspergillus* section *Flavi*, GeoThi *Fusarium* sp.), h: Fungi from oil environment (FL10 *Penicillium* section *Citrina*, FL6 *Aspergillus* section *Terrei*), i: Fungi from hot springs (FH3 *Aspergillus* section *Fumigati*, FH101 *Aspergillus* section *Nidulans*), all on PDA, 5–6 days.

Comparing all samples species of *Aspergillus* had the highest prevalence in our collection with 69 samples corresponding to 42% of the library (from 164 sequenced cultures), followed by species of *Penicillium* with 20 samples (12.2%) as well as species of *Trichoderma* with 20 samples (12.2%) and species belonging to the genus *Fusarium* with 14 samples (8.5%). Representative strains of this collection can be found in Fig 2, while the whole collection is outlined in S1 Fig.

### Enzymatic analysis

To facilitate a prescreening of the enzymatic activity, agar plate tests with supernatant of fungi growing in inductive media were conducted. With this plate based screening the number of fungal strains to be investigated in detail decreased to 69 (42% of the 164 tested strains). Productive strains were identified in all habitats, e.g. wood (17), soil (6), rice straw (3), fruits (2), oil environment (7), hot springs (4), insect shells (18), shrimp shells (11), and crab shell (1). For 10 strains activities were lower than the detection limit in the quantitative assays.

**Fig 3:**
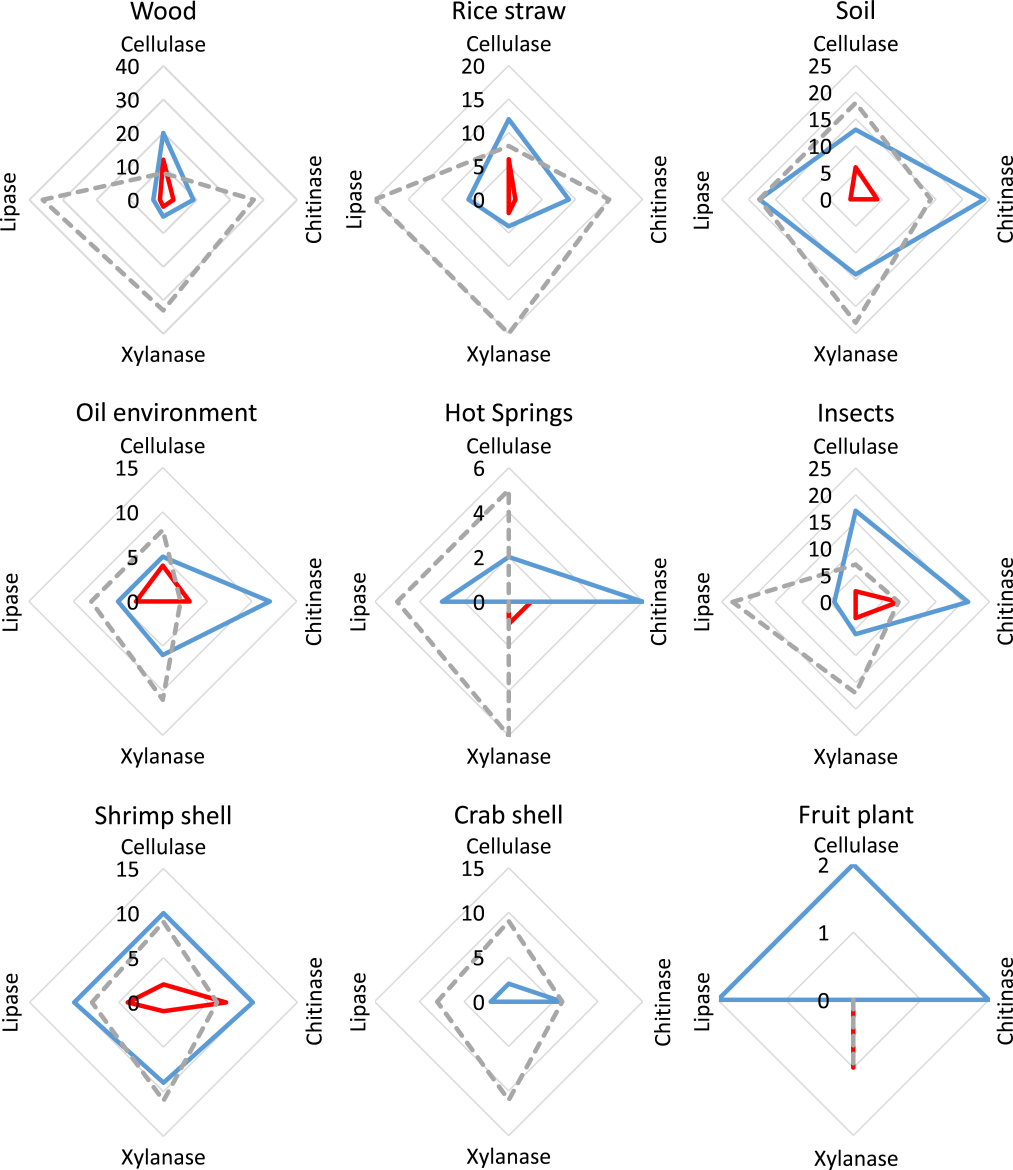
Enzymatic activity of the fungal collection in plate-based screening. All activity estimations are based on substrate degradation by fungal in plate-based screening. Number of strains with high activity is shown in red, low activity is shown in blue and activity below detection limit as light grey line.

Over 85% of the strains (59 of 69) had a high activity for at least one of the investigated enzyme activities (Fig 3). Most strains with high cellulase activity originated from woody substrates, rice straw and soil. Interestingly, a weak cellulase activity was also detected in a number of species originating from shrimp shells and insects, which might point to weak unspecific beta-glucoronidase activity. Similar effects can be seen in the case of chitinase activity (Fig 3). We expected a high number of fungal strains with chitinase activity from shrimp shells and insects due to the high amount of chitin and we could confirm the activity, but found additionally strains from hot springs, oil environments, soil, rice straw and wood. A lower number of active strains with lipases and xylanases were found. High activity for lipase activity was found on oil environment and on shrimp shells, due to high occurrence of high molecular weight hydrocarbons in these environments but lower activity was more widely distributed and found in all habitats peaking at soil (18 strains). Xylanase activity was, similarly distributed as cellulase activity, due to the co-existence in lignocellulose substrate. The activity was, as expected, peaking at wood, rice straw and soil but had also substantial activity in fungi isolated from insects and shrimp shells. These results point out, that the specific activities fit well to the supposed activity which is needed to degrade the substrates of the corresponding environment.

Cellulase activity was the most often identified one and 18 of the 44 cellulase producing strains were solely capable of growing on cellulose as primary carbon source. The best cellulase producers belong to *Aspergillus* section *Terrei* like SF31 and *Fusarium* spp. like Fsh13 and FW16.1, all showing an activity of 0.1 U/mg. From the 24 xylanase producing strains 3 were growing on xylan only and 9 strains were capable of utilizing both xylan and cellulose. A similar distribution was observed when cellulase and chitinase activity was compared. Next to the 18 strains, degrading only cellulase and described above, 7 strains were solely chitinase positive and 7 other strains with dual activity were found.

Fungal strains of high chitinase activity belong to *Humicola*, *Fusarium* and *Aspergillus* section *Fumigati*, *Nidulans* and *Flavi*. Especially the *Aspergilli* were good producers, as seen by Fsh101 (*Aspergillus* section *Nidulans*) with 0.13 U/mg, FF1 (*Aspergillus* section *Flavi*) with 0.13 U/mg and FW35 (*Aspergillus* section *Fumigati*) with 0.15 U/mg activity. However, the low number of isolates with high chitinase activity (9) is in contrast to the number of isolates from the chitin containing habitats (in total 87), insect shells, shrimp shells and crab shells.

Fungal strains showing high xylanase activity (13) could be isolated from insects, oil environment, rice straw, crab shell, wood and soil environments (from in total 253). They belong to the genus *Talaromyces*, *Aspergillus* section *Nidulans* and *Flavi* and *Penicillium* section *Citrina*. Fsh17 and FR1 (both *Aspergillus* section *Flavi*) with 0.74 U/mg and 0.97 U/mg and FL100 (*Penicillium* section *Citrina*) 1 U/mg, showed the highest xylanase activities.

**Fig 4:**
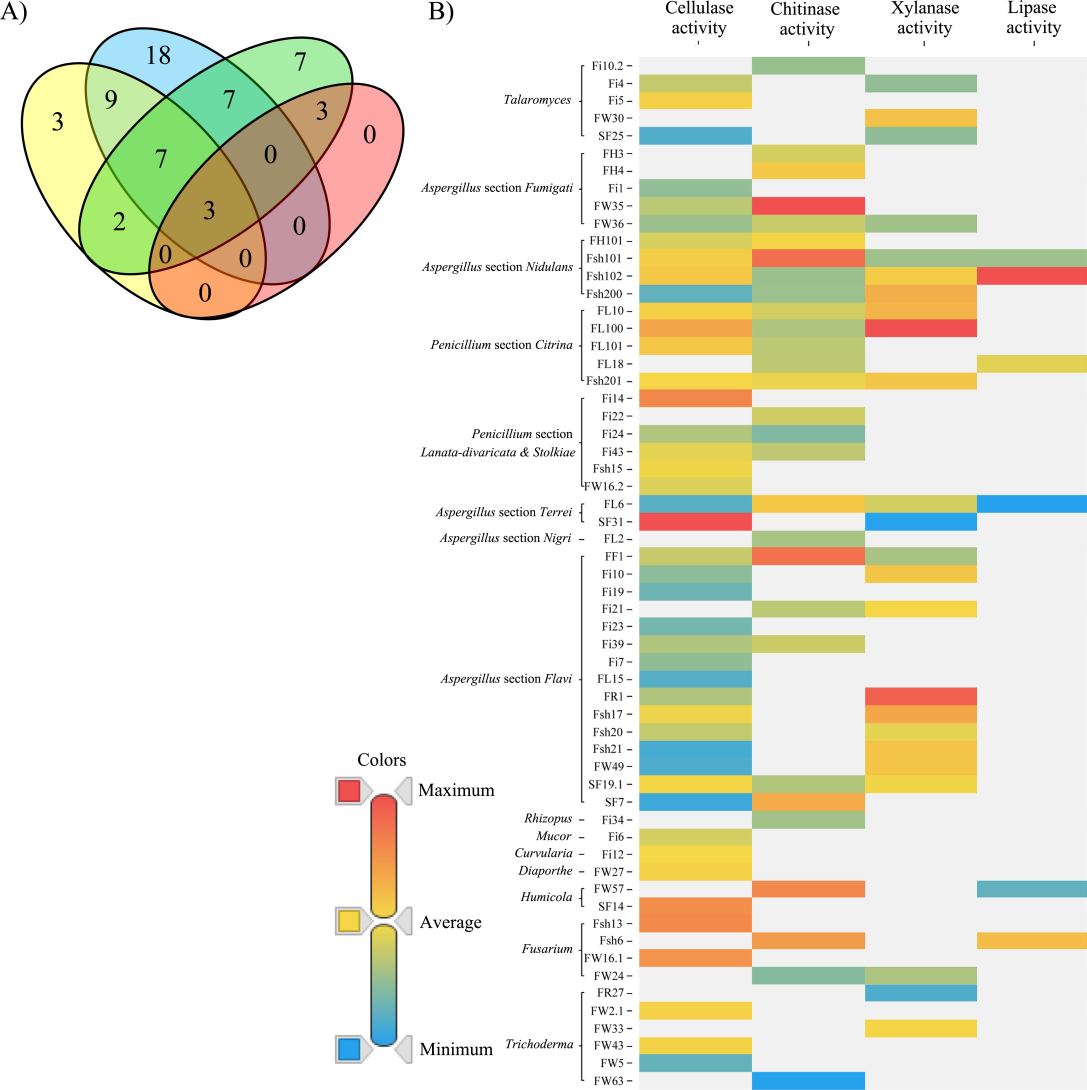
Enzymatic activities of the most productive fungal strains. (A) Number of strains with cellulase (blue), chitinase (green), lipase (red), and xylanase activity (yellow), measured in the supernatant. (B) Different specific enzymatic activities (U/mg) of fungal isolates sorted by their respective taxonomic position (Fig 1). Red, high activity; yellow, average activity; blue, low activity. Values are chitinase 0.15, 0.05 and 0.00 U/mg, lipase 0.27, 0.12 and 0.04 U/mg, cellulase 0.09, 0.02 and 0.00 U/mg and, xylanase 1.03, 0.58 and 0.23 U/mg, for high, average and low activity, respectively.

A small number of fungal strains with high lipase activity (2) were isolated from the oil environment and shrimp shells (51 isolated strains) and belong to *Fusarium* and *Aspergillus* section *Nidulans*. Specifically, the strains FL18 (*Penicillium* section *Citrina*), with 0.11 U/mg, Fsh6 (*Fusarium* sp.) with 0.14 U/mg and Fsh102 (*Aspergillus* section *Nidulans*) with 0.27 U/mg activity were the most prolific producers. An overview of the enzymatic activities can be found in Fig 4B. From a biotechnological point of view, producers with a high activity in all four investigated enzymatic activities are especially interesting (Fig 4A). It was found, that 7 candidates showed activities for the degradation of three different materials, while remarkable 3 are able to use all four investigated carbon sources. These high performance degraders belong in the *Penicillium* section *Citrina* and *Aspergillus* section *Nidulans* clades, showing activity in the most cases for three or all four investigated activities. In general, fungal strains of the *Trichoderma* taxa, performed relatively poorly, as they only showed low-to-medium activities in one of the four tests performed and no activity for the other three.

## Discussion

By looking for new candidate strains and enzymes for the biotechnological conversion of natural waste products into value added products, more than 200 fungal strains have been screened for their secreted cellulase, chitinase, xylanase, and lipase activity. These fungal isolates originate from different promising habitats and locations in Vietnam, a country with a high level of biodiversity and a treasure for yet unknown microbial resources.

Most of the fungal strains have been affiliated with the Ascomycota genera *Aspergillus*, *Penicillium*, *Trichoderma* and *Fusarium*, highlighting the fact that these fungi occur in various habitats around the world [48–51]. The dominance of these fungi was already observed in other cultivation attempts [52]. The minority of the strains (20%) have been affiliated to *Mucor*, *Rhizopus*, *Cunninghamella* and *Gongronella* (Mucoromycota), and *Humicola*, *Taifanglania*, *Diaporthe*, *Lasiodiplodia*, *Curvularia*, *Talaromyces* (Ascomycota). Strains affiliated to *Taifanglania*, *Lasiodiplodia*, *Cunninghamella* and *Gongronella* had no significant secreted enzyme activity, in tests of this study. Members of these fungal taxa have rarely been described as cellulose, xylan and chitin degraders. In general observed enzyme activities, corroborated the substrate composition of the habitats.

While attempting to isolate fungal strains with a reasonable chitinase activity from shells of insects, shrimps and crabs, only two strains with high chitinase activity originated from shrimp shells. Interestingly, four strains (*Humicola*, *Aspergillus* section *Fumigati* and two strains *Aspergillus* section *Flavi*) with high chitinase activity originated from habitats such as wood, fruits and soil. Fungal chitinases are described from different *Aspergillus*, *Penicillium* and *Fusarium* species as well [53–55]. We did not isolate *Trichoderma* strains with high chitinase activity, as shown by previous studies [53,54], however the *Trichoderma* from this collection strains were isolated from rice straw and wood, exhibit a xylanase and cellulase activity of 0.58 U/mg and 0.022 U/mg, respectively. A noticeable fact is that species of *Trichoderma* are underrepresented in the chitin based-samples (0 crabs and shrimp shells and 2 (6.7%) in insects) of this collection. They are more abundant in the wood samples with 27% and soil with 27.3%, again this is in line with others studies identifying also a high percentage in soil and wood samples [56].

The *Aspergillus* strains Fsh101 (*Aspergillus* section *Nidulans*), FF1 (*Aspergillus* section *Flavi*) and FW35 (*Aspergillus* section *Fumigati*) had a specific chitinase activity of 0.13 U/mg, 0.13 U/mg, 0.15 U/mg respectively, comparable with the chitinase activity 0.24 U/mg of *Aspergillus nidulans* [57]. The CAZy database lists for *Aspergillus nidulans* FGSC A4 19 genes encoding for glycoside hydrolase of family GH18 (10 GH18, 9 GH18+CBM18/CBM50, for *Aspergillus niger* CBS 513.88 14 GH18 (9 GH18, 5 GH18+CBM18/CBM50), and *Aspergillus oryzae* RIB40 18 GH8 (15 GH18, 4 GH18+CBM18/CBM50). However, the specific activity of these chitinases differs considerably, as shown for two chitinases ChiB1 and ChiA1 (GH18) from *Aspergillus fumigatus* ATCC 13073 with 0.94 U/mg (with 0.5% chitin) [58] and 2x10^^-4^ U/mg (calculated according to Rush et al. with 300 mM substrate corresponds to 0.25%) respectively [59]. A chitinase from *Trichoderma viride* with a specific activity of 600 U/mg is available as commercial product (Sigma Aldrich, order numbers C8241, Germany). This high activity may have been achieved by different modifications, such as optimization of expression conditions, purification and mutagenesis as indicated by Omumasaba et al. using a *Trichoderma viride* strain with an original chitinase activity of 0.1 U/mg [60]. Currently, chitinases are applied for the activation of plant defense by using active chito-oligosaccharides [61] as well as the biocontrol of plant pathogenic fungi (degradation of the fungal cell wall) [62] and insects (degradation of the insect exoskeleton) [63].

The strains Fsh13 (*Fusarium* sp.), FW16.1 (*Fusarium* sp.) and SF31 (*Aspergillus* section *Terrei*) had the highest cellulase activity with 0.1 U/mg. Members of the genus *Fusarium* are common in plants as endophytes and pathogens and have been described as producers of active cellulases [64]. *Fusarium oxysporum* and *Aspergillus terreus* were described with a slightly more effective cellulase with a specific activity of 0.9 U/mg [65], and 0.56–0.85 U/mg, respectively [66,67]. By checking the CAZy database only one putative cellulose (GH5) from *Fusarium solani* (more related to FW16.1) was found [68]. Different *Fusarium oxysporum* species (more related to Fsh13) expressing different cellulases. The *Fusarium oxysporum* KCTC 16909 has one GH1, a β-glucosidase, [69] with 268 U/mg for the purified enzyme. Another *Fusarium oxysporum* [70] has at least two GH7, endoglucanases Cel7A [70] and Cel7B also known as endoglucanase I (PDB code 1OVW) [71]. For *Aspergillus terreus* SUK-1 different cellulases are listed in the CAZy, one putative GH3, β-glucosidase, (Genbank accession number ACY03273), one putative GH5, an endoglucanase, (Genbank accession number AAW68436) and one putative GH7, a cellobiohydrolase, (Genbank accession number AAW68437). Commercially available cellulases can be in a range with similar activity like the homologous expressed *Aspergillus niger* cellulase lyophilized powder (Sigma Aldrich, order numbers C1184 and 22178, Germany) with 0.3 U/mg and 0.8 U/mg, respectively. These enzymes are used in animal feed [72], food [73], textiles [74] and in the paper industry [75]. An interesting observation was, that also species of *Trichoderma* are known to process cellulases [21,76] and xylanases [77] of high activity, we found cellulases with high activity in *Penicillium* section *Citrina* and xylanases with high activity in *Aspergillus* section *Flavi*, but *Trichoderma* species of this study had only minimal to moderate cellulase and xylanase activities. Strains Fsh17, FR1 (0.74 U/mg and 0.97 U/mg, *Aspergillus* section *Flavi*) and FL100 (1 U/mg, *Penicillium* section *Citrina*) had a specific xylanase activity in the lower range compared to xylanases of *Aspergillus flavus* with 0.11–20.40 U/mg [78]. Xylanases of *Penicillium citrinum* FERM P-15944 / MU-4 with 1.8 U/mg (GH10) [79] or 1.74 U/mg (GH11) [80] are listed in the CAZy database. These enzymes could reach a specific activity of 70 U/mg or 303 U/mg, respectively. Commercially available xylanase of *Trichoderma longibrachiatum* with 1 U/mg (Sigma Aldrich, order numbers X2629, Germany) or xylanase of *Aeromonas punctata* with 8.4 U/mg is available (Megazyme, order numbers E-XYNAP, Ireland) are available. Beside biofuels, xylanases are used for other various industrial processes such as food, feed, and pulp and paper industries [81]. Current applications are pre-bleaching pulp to reduce the chemical bleaching [82], increasing loaf volumes during baking [83], enhancing the weight gain of broiler chickens by incorporation of xylanase into the rye-based diet food [84], clarifying juices of fruits and vegetables [85], and clearing the wastewater from agricultural waste like corn cobs [85].

The high lipase activity of the strains *Fusarium* sp. Fsh13 (0.14 U/mg) and *Aspergillus* section *Nidulans* sp. Fsh102 (0.27 U/mg) was in the range of the previous descripted lipases from *Fusarium* sp. YM-30 with 0.15 U/mg [86] and *Aspergillus nidulans* lipases [87] with 0.3 U/mg. Both strains originated from shrimp shells which contain a significant amount of high molecular weight hydrocarbons as a substrate for these enzymes. Commercial lipases exihibit similar activities like the lipase from *Aspergillus* sp. with 0.2 U/mg (Sigma-Aldrich, order number 84205, Germany) or from *Candida antartica* with 0.3 U/mg (Sigma-Aldrich, order number 02569, Germany). Lipases application scope ranges from biodiesel production [88], over food and nutraceutical industries [89] to detergent industry [90].

Very diverse in their enzymatic spectra are the fungi from oil environments, FL6 (*Aspergillus* section *Terrei*), FL10 and FL100 (both *Penicillium* section *Citrina*) (3 from 7, 42%) and shrimp shells Fsh101, Fsh102, Fsh200 (all *Aspergillus* section *Nidulans*) and Fsh201 (*Penicillium* section *Citrina*) (4 from 10, 40%), as well as one strain isolated from fruits FF1 (*Aspergillus* section *Flavi*, 1 of 2, 50%), one strain from wood FW36 (*Aspergillus* section *Fumigati*, 1 of 17, 6%) and one isolate from soil SF19.1 (*Aspergillus* section *Flavi*, 1 of 6, 17%), showed activity in at least three of the analyzed enzymatic activities. The strains FL6 (*Aspergillus* section *Terrei*), Fsh101 and Fsh102 (both *Aspergillus* section *Nidulans*) showed activities in all four investigated assays. It seems that fungal strains of these two environments (oil environments and shrimp shells) have a wider enzymatic spectrum in comparison to strains of other habitats. Both environments have a heterogenic composition, while shrimp shells consist of chitin backbone, protein parts and lipids, the composition of oil environments consists of lipids and proteins. Maybe the heterogeneity of different energy sources causes diversity of enzymatic toolbox of the strains, which is needed for efficient degradation of substrates found in these habitats.

A key feature of profitable high performance degraders is a low substrate or product inhibition. This grants the possibility to use high amount of substrate, while getting high quantities of product as it was shown already for some secreted fungal cellulases and xylanases [91]. However, the two major limitations of the known cellulases in heterologous systems are that either truncated enzymes with low or no activities are expressed or hyperglycosylation occurs, which also diminishes enzyme activity [92]. The enzymes presented in this study could be new targets to overcome these limitations of nowadays used enzymes, if they are heterologously expressed. Varying cultivation conditions may increase the specific enzyme activity as has been shown for *Aspergillus flavus* and *Aspergillus niger* up to 200 fold [78]. Beside different growth media another option to increase the enzymatic activity might be a solid-state fermentation as has been shown for *Penicillium citrinum* were increased the specific xylanase activity could be improved up to 150 fold [93,94]. Moreover for industrial applications several steps have to be reached: firstly, the use of purified or heterologously expressed enzymes to show their high performance capacity, while in the second step promising enzyme candidates have to be mutagenized to complete the whole concept for improved biocatalyst. Enhancing applicability by purification was shown for cellulases of a *Fusarium oxysporum* strain which increased the specific activity 30 fold [65], for chitinases of *Aspergillus fumigatus* S-26 increasing their activity 61 fold [95], for xylanases from a *Penicillium citrinum* isolate increasing the specific activity 23 fold [96] and lipases from *Fusarium* sp. YM-30 [86] and *Aspergillus nidulans* [87] whose activity could be increased several hundred fold. We used the same standard growth conditions for all tested 221 strains, to ensure comparability within the screen. It is likely, that by evaluation of the optimal conditions (media, temperature, light regimen) the various enzyme activities can be increased. Additionally, different substrates like fatty acids of different chain length for lipase activity or different celluloses for cellulase activity can be tested. If considered that, high performance strains of this study could be identified by using standard growth conditions the existing potential of this strain collection could be exhausted when optimized enzyme conditions are used for enzyme production. For the 221 fungal strains four different substrates were used, but others interesting activities could be investigated like polyethylene terephthalate (PET) degrading enzymes [97] to reduce the plastic waste, amine transferring enzymes for fine chemicals [98] or active pharmaceutical ingredients [99,100] production. The high potential of this fungal collection is now made available for the public.

## Conclusion

In this study the identification of 295 novel fungal isolates from nine different substrates and environments from the biodiverse country Vietnam is reported. From these, 59 strains demonstrated the capability to degrade cellulose, chitin, xylan or triglycerides. High performance degraders could be identified on all four polymeric substrates. In general, the enzymatic activities pattern matches very well to the habitats they were isolated from. The potential of *Aspergillus* and *Penicillium* strains as high performance degraders is highlighted. Moreover, we accomplished to isolate strains with high cellulase, xylanase and chitinase activity, within taxa that have not been recognized as important candidates for biotechnology, e.g. *Curvularia*, *Diaporthe* and *Talaromyces*. These results indicate a linkage between the habitat of the isolated fungi and their genetic disposition of degrading enzyme activities. In future searches of specific enzyme activities the environment of the collected microorganisms should be taken into consideration to increase the probability of a high degradative capability.

## Supporting information

S1 Fig Pictures of the species of the fungal collection. (PDF)

S2 Fig Plate assay. (PDF)

S3 Fig Phylogenetic tree fungal strains without Aspergillus / Penicillium / Talaromyces. (PDF)

S4 Fig Phylogenetic tree fungal strains only Aspergillus / Penicillium / Talaromyces. (PDF)

S5 Fig Map of Vietnam with sample places. (EPS)

S1 Table Table of DSMZ ID, geographic origin, habitat origin, and relative enzyme activities. (XLSX)

S2 Table Table of enzymatic activity. (XLSX)
