# Supplementary material for: A unique fungal strain collection from Vietnam characterized for high performance degraders of bioecological important biopolymers and lipids
Source: PLoS One. 2018 Aug 30;13(8):e0202695. doi: 10.1371/journal.pone.0202695 (PMC6117010; doi:10.1371/journal.pone.0202695)
Supplement: S1 Fig — (PDF) [file pone.0202695.s001.pdf]

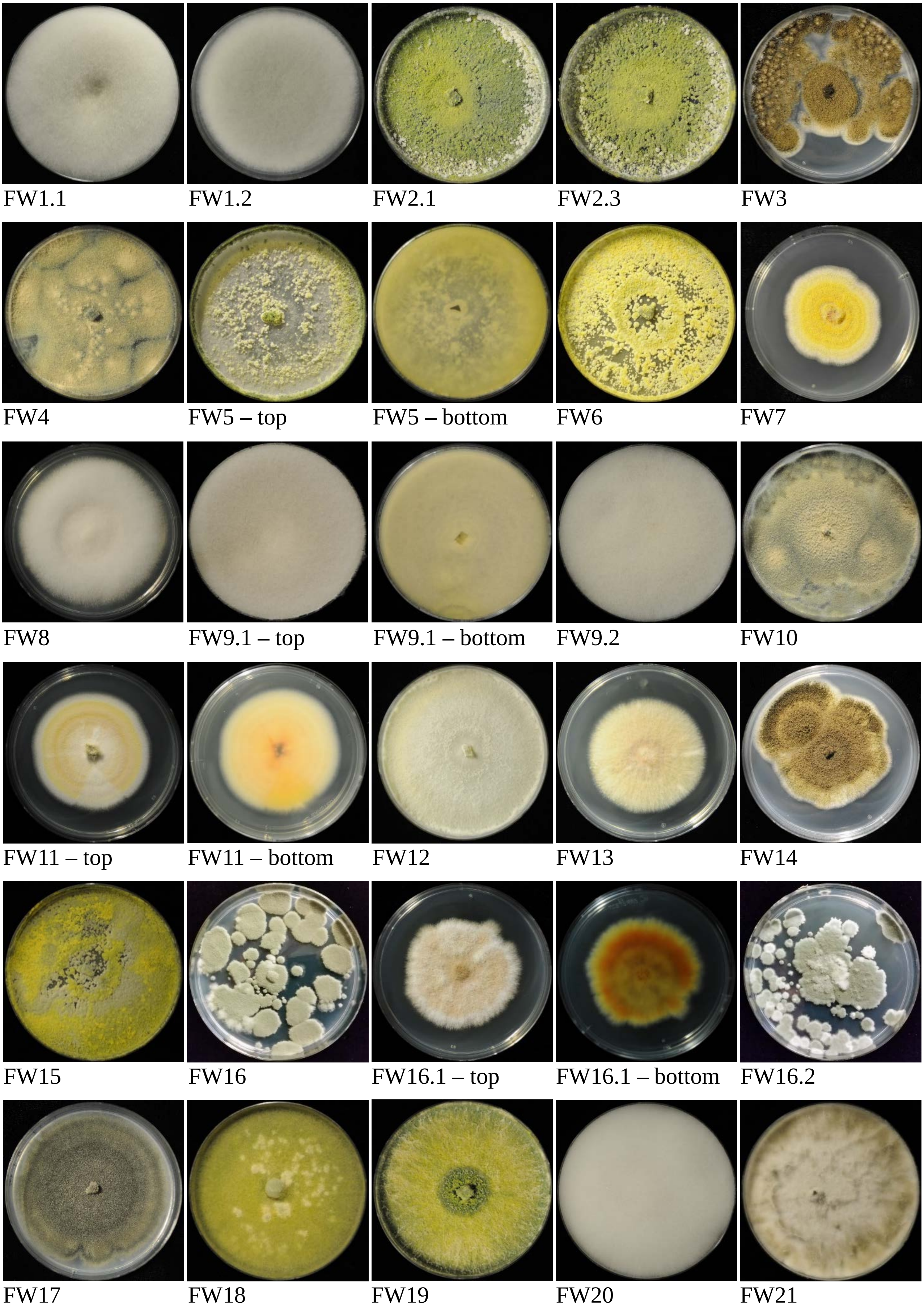

Supporting Figure 1-1: Phenotypic characterization of fungi collected from wood. If coloring on top compared to bottom of the mycelium was different both views are presented.

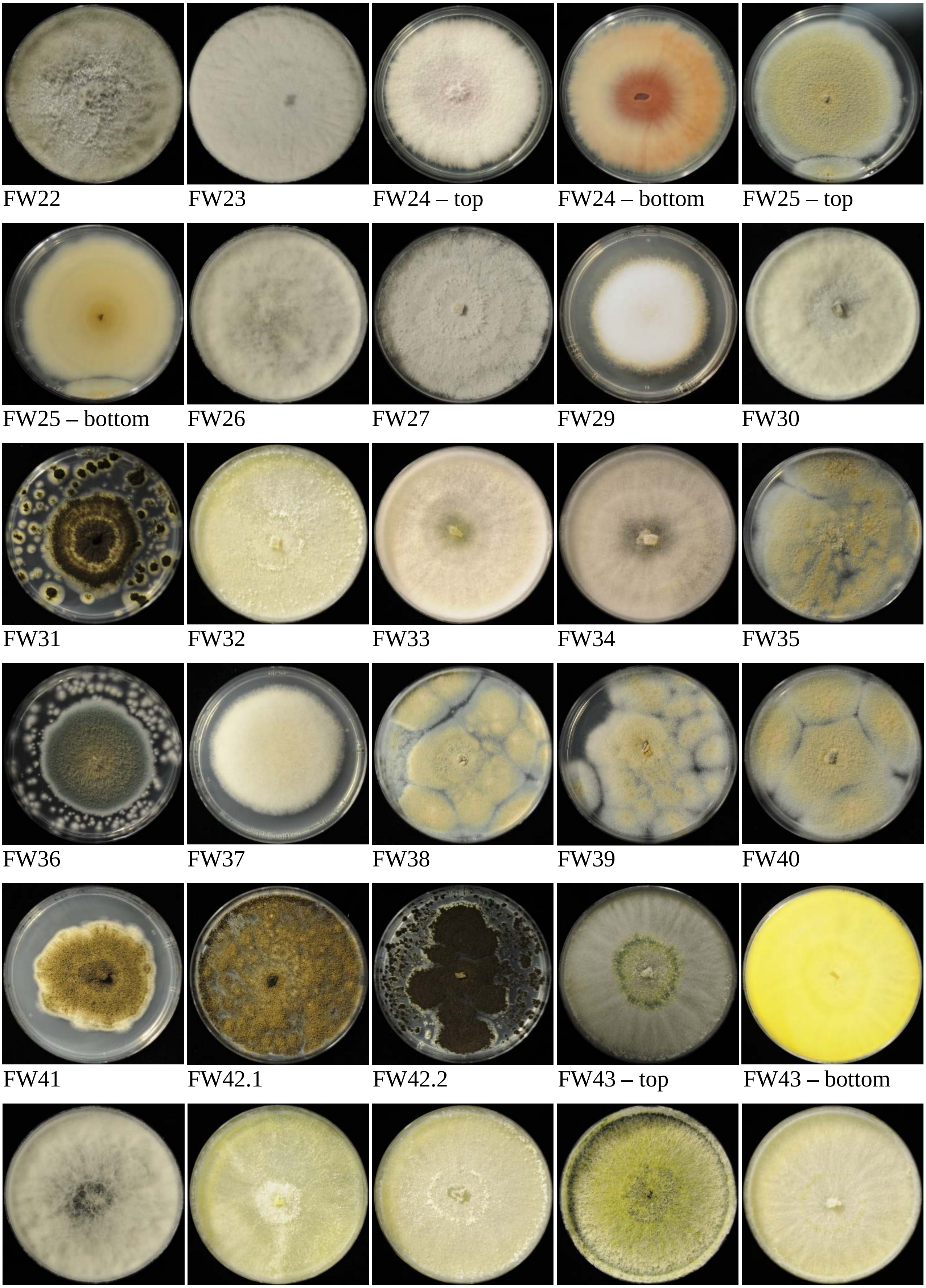

Supporting Figure 1-2: Phenotypic characterization of fungi collected from wood. If coloring on top compared to bottom of the mycelium was different both views are presented.

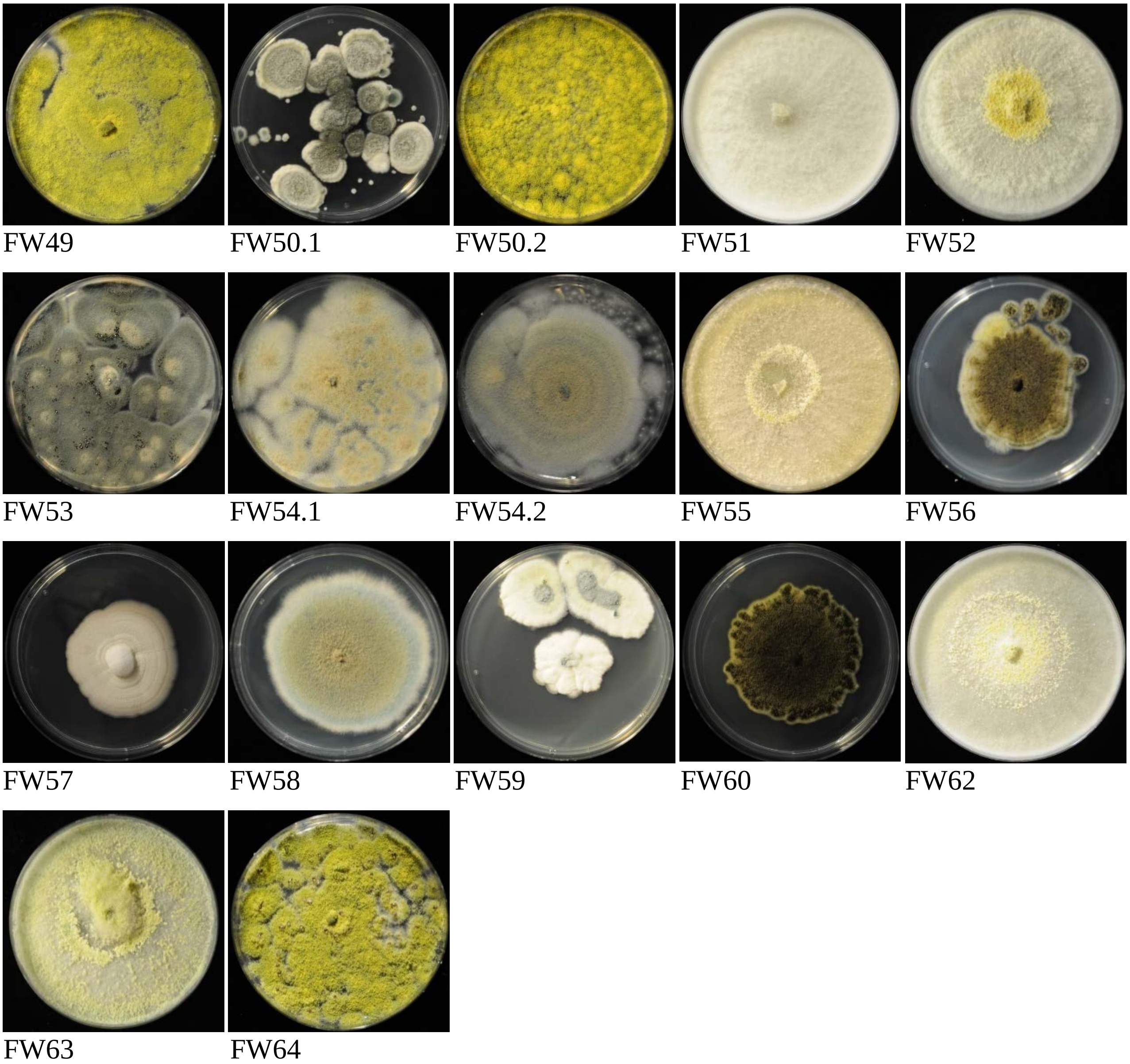

Supporting Figure 1-3: Phenotypic characterization of fungi collected from wood. If coloring on top compared to bottom of the mycelium was different both views are presented.

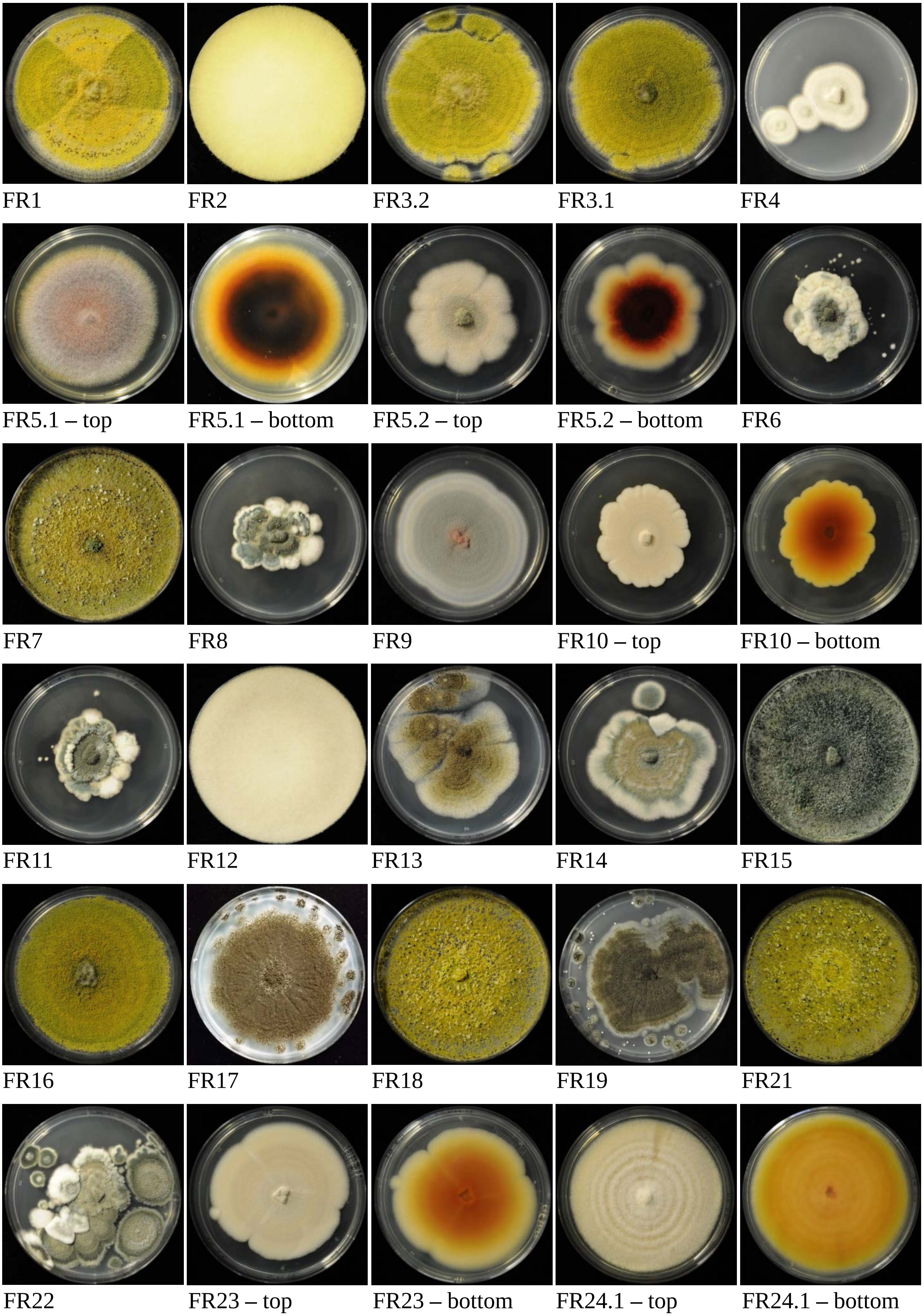

Supporting Figure 1-4: Phenotypic characterization of fungi collected from rice straw. If coloring on top compared to bottom of the mycelium was different both views are presented.

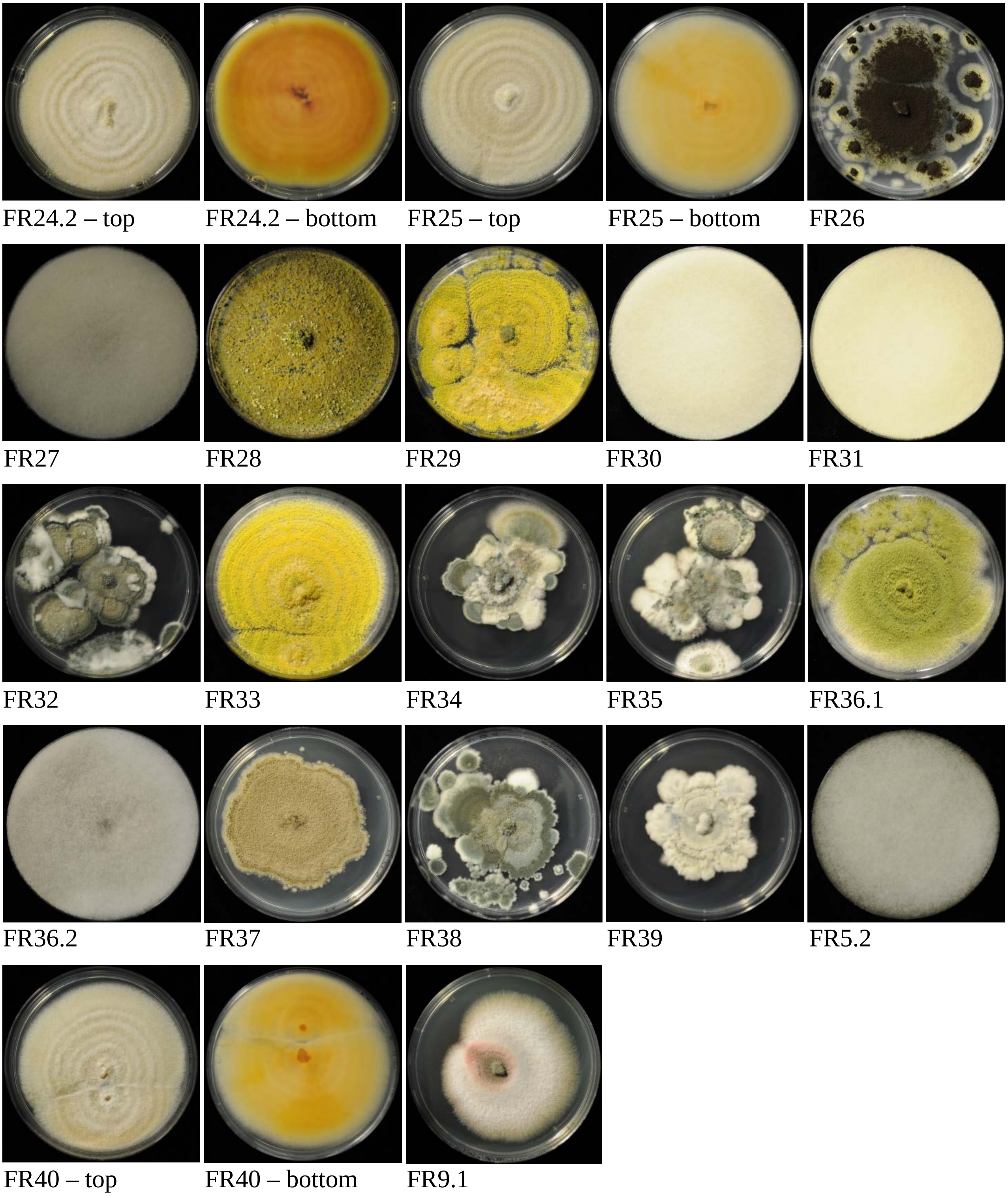

Supporting Figure 1-5: Phenotypic characterization of fungi collected from rice straw. If coloring on top compared to bottom of the mycelium was different both views are presented.

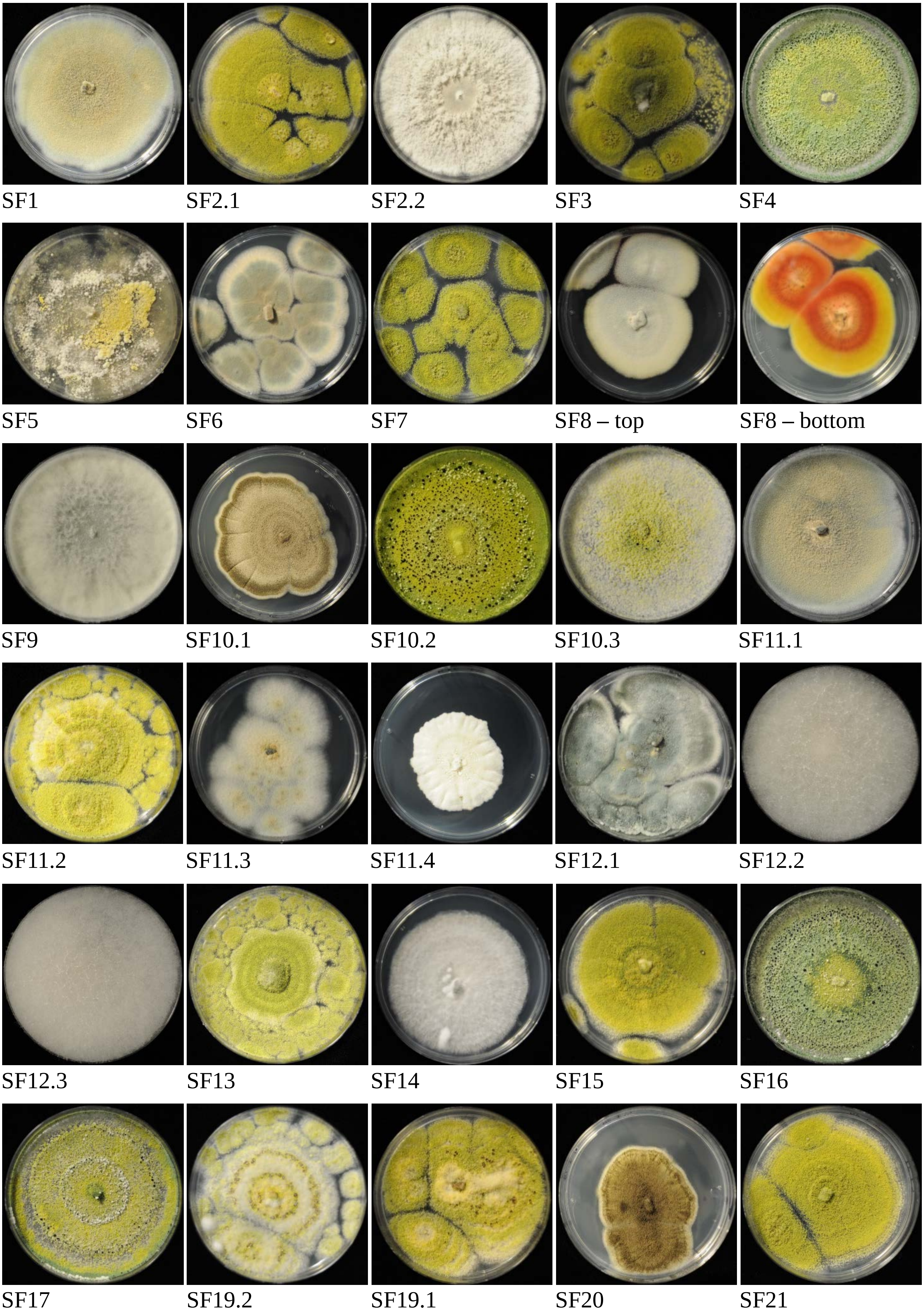

Supporting Figure 1-6: Phenotypic characterization of fungi collected from soil. If coloring on top compared to bottom of the mycelium was different both views are presented.

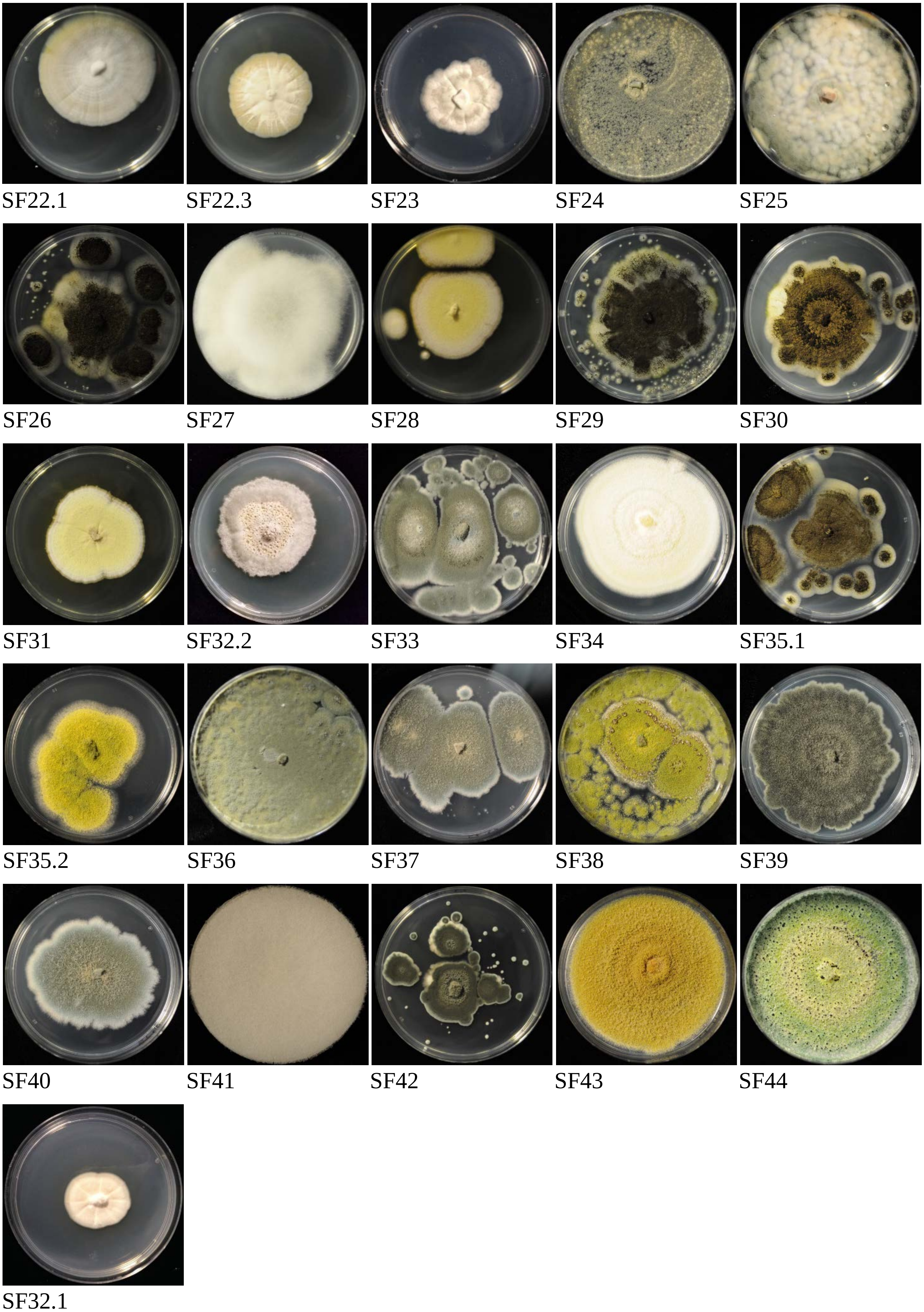

Supporting Figure 1-7: Phenotypic characterization of fungi collected from soil. If coloring on top compared to bottom of the mycelium was different both views are presented.

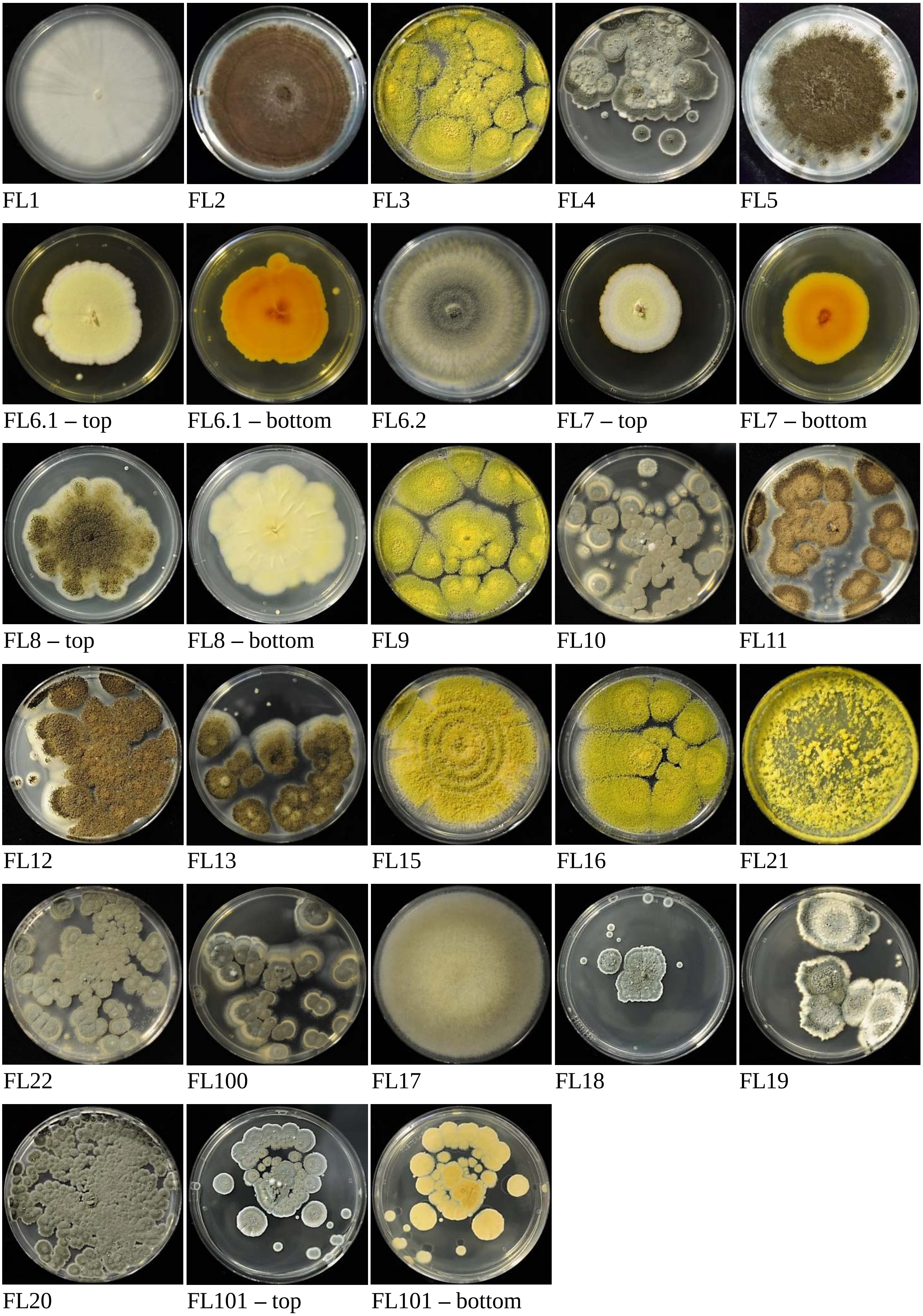

Supporting Figure 1-8: Phenotypic characterization of fungi collected from oil environment. If coloring on top compared to bottom of the mycelium was different both views are presented.

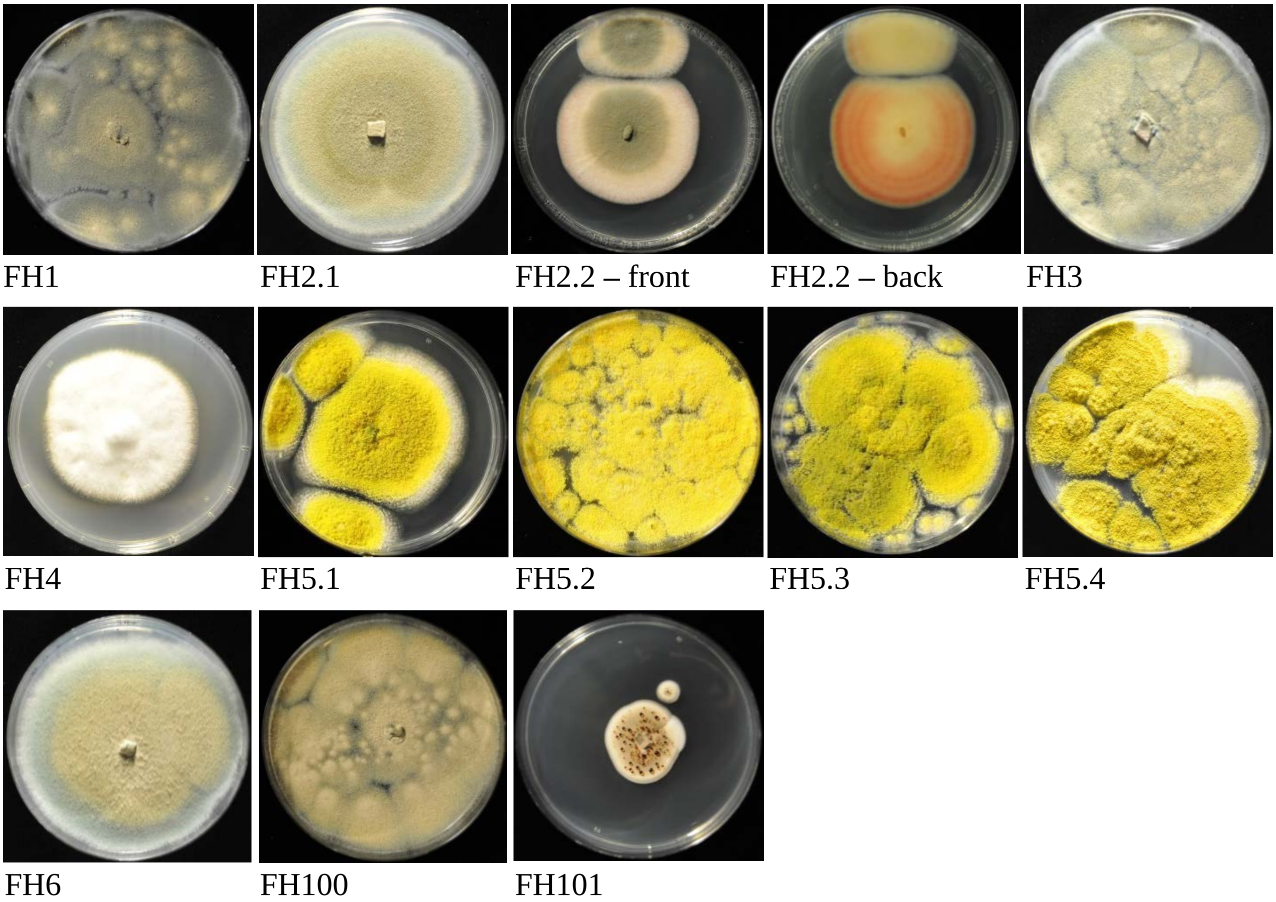

Supporting Figure 1-9: Phenotypic characterization of fungi collected from hot springs. If coloring on top compared to bottom of the mycelium was different both views are presented.

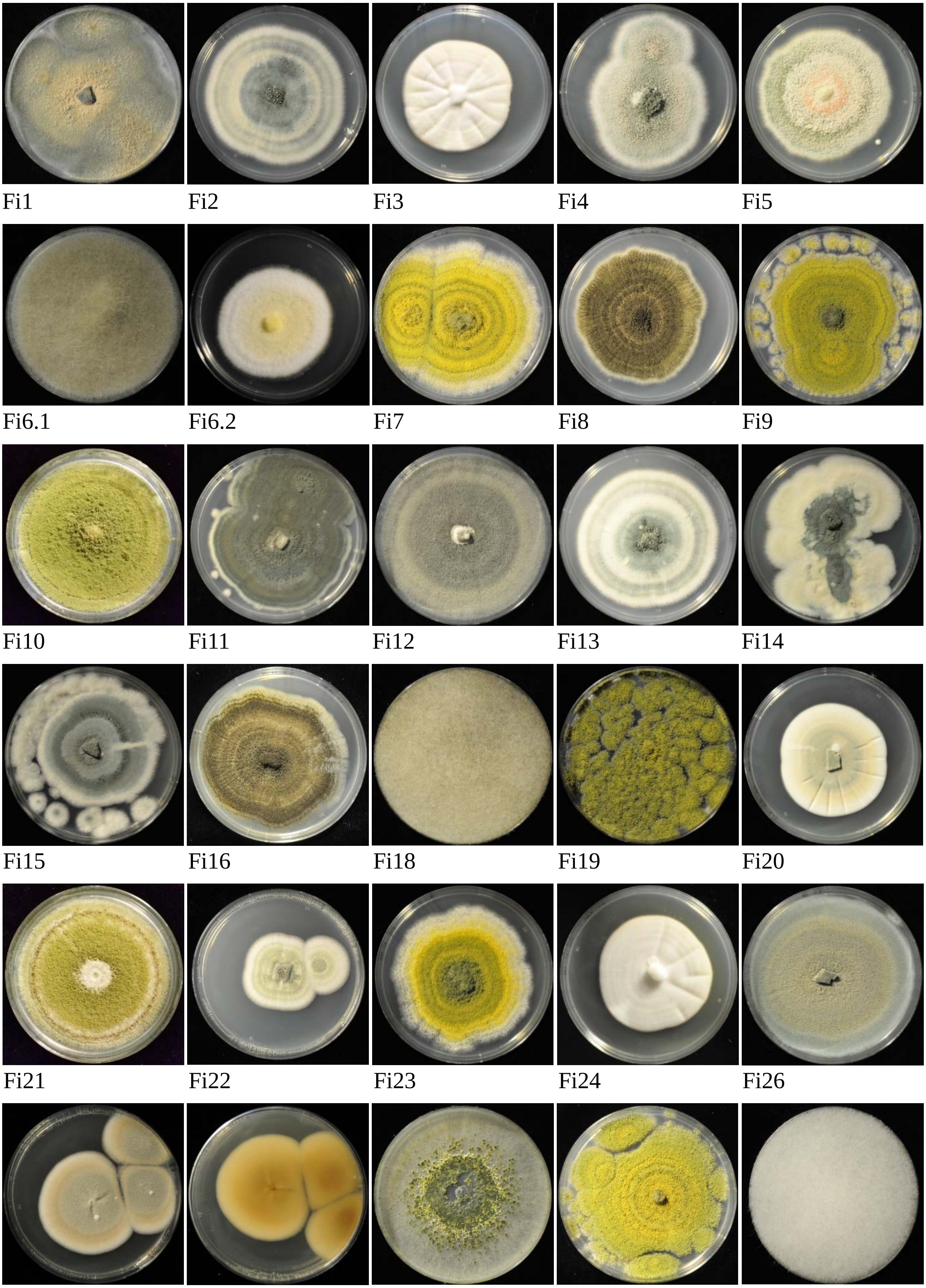

Supporting Figure 1-10: Phenotypic characterization of fungi collected from insects. If coloring on top compared to bottom of the mycelium was different both views are presented.

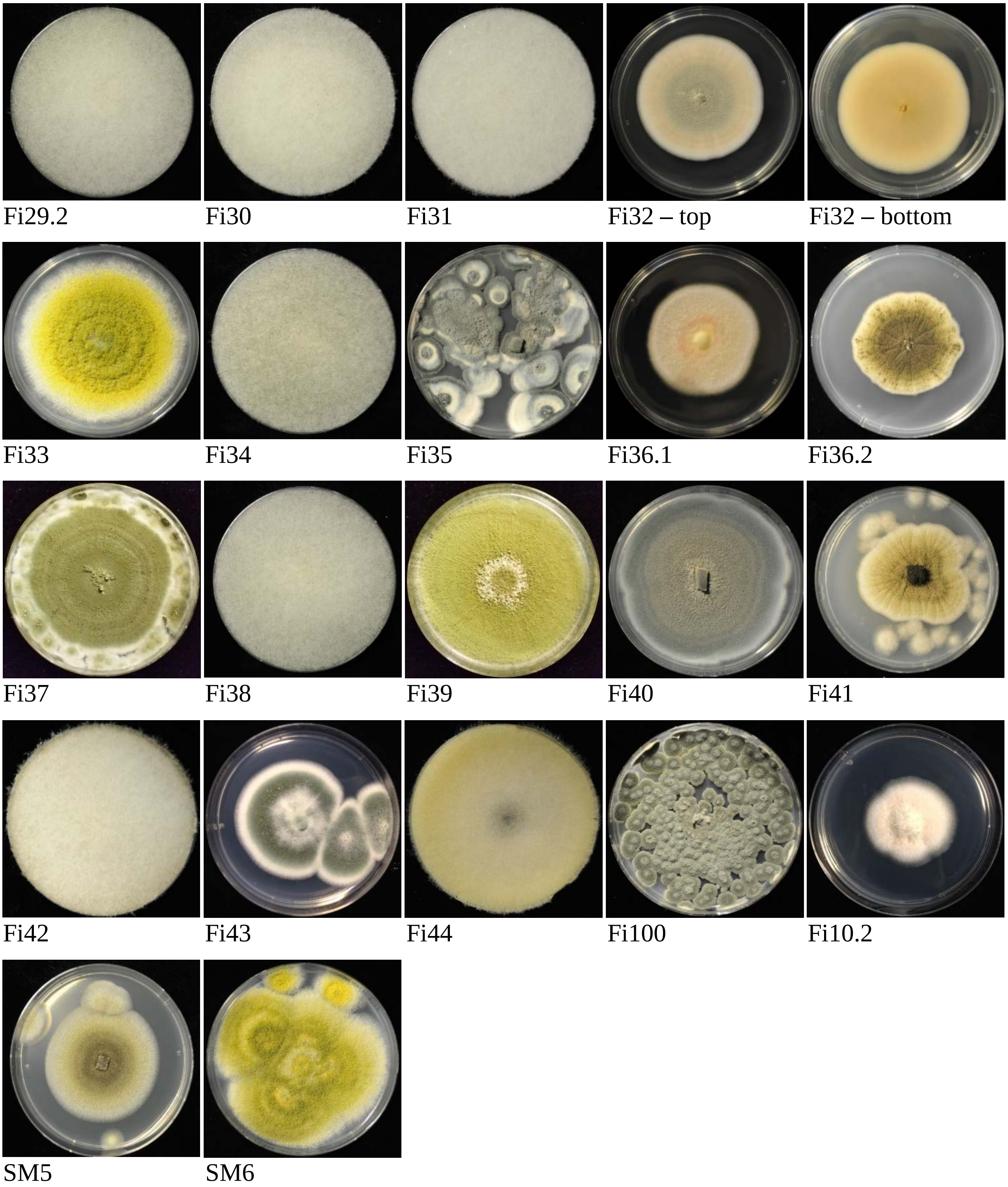

Supporting Figure 1-11: Phenotypic characterization of fungi collected from insects. If coloring on top compared to bottom of the mycelium was different both views are presented.

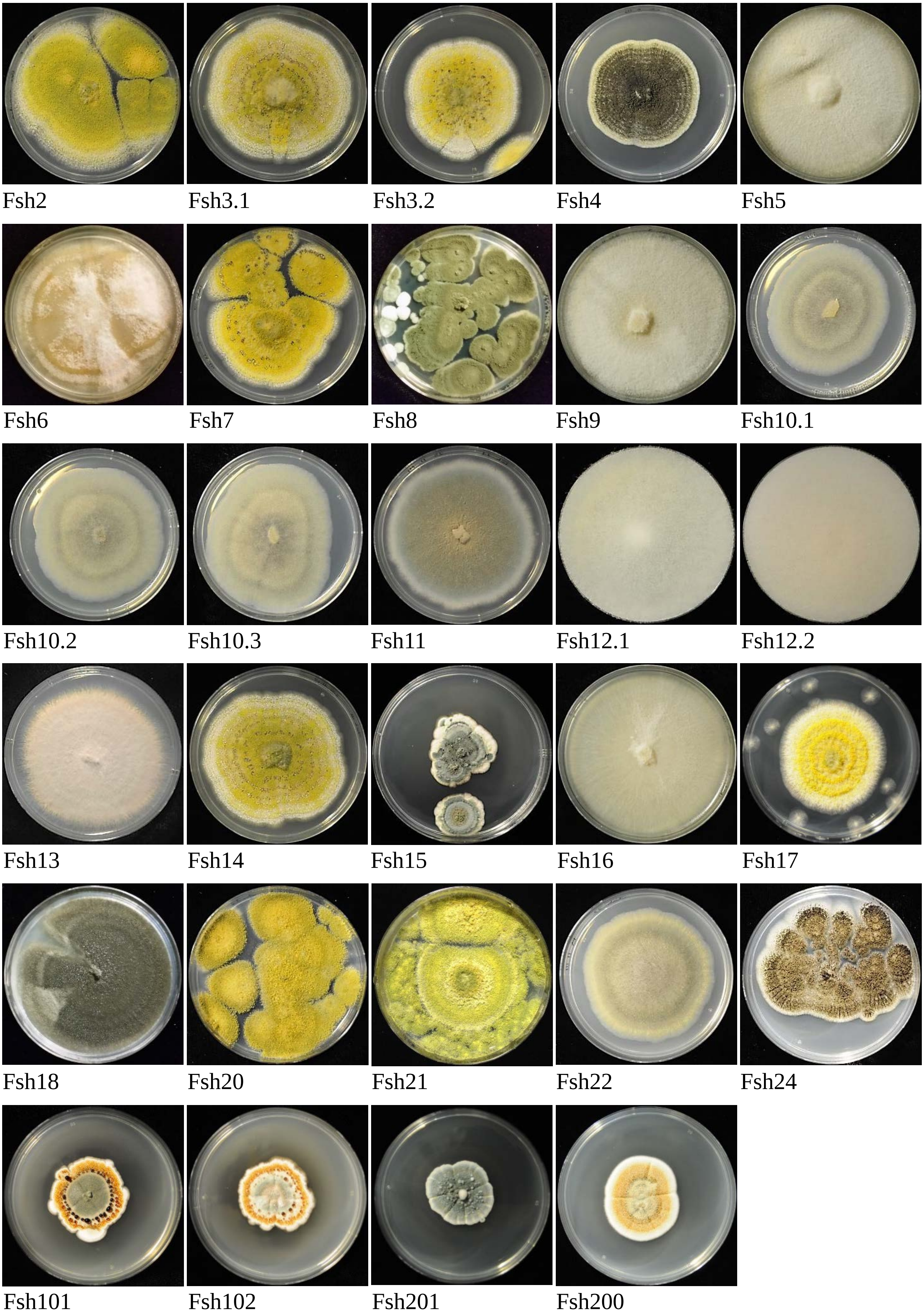

Supporting Figure 1-12: Phenotypic characterization of fungi collected from shrimp shells. If coloring on top compared to bottom of the mycelium was different both views are presented.

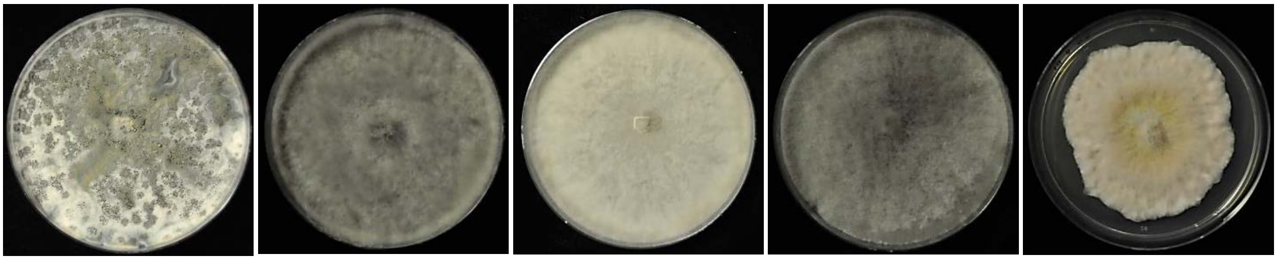

FC1 FC2 FC3 FC4 FC5

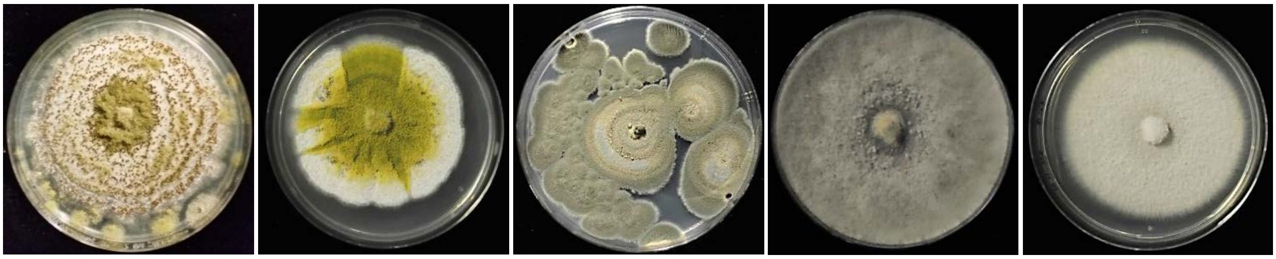

FC6.1 FC6.2 FC7 FC8 FC9

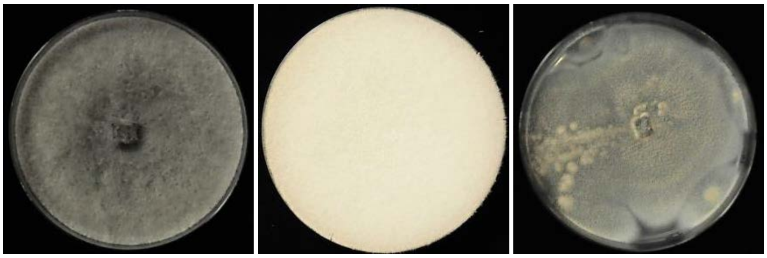

FC10 FC11 FC12

Supporting Figure 1-13: Phenotypic characterization of fungi collected from insects. If coloring on top compared to bottom of the mycelium was different both views are presented.

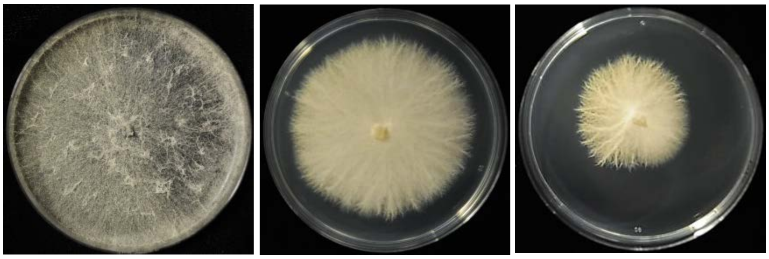

FF1 FF2 GeoThi

Supporting Figure 1-14: Phenotypic characterization of fungi collected from fruit plants. If coloring on top compared to bottom of the mycelium was different both views are presented.
