# Supplementary material for: A unique fungal strain collection from Vietnam characterized for high performance degraders of bioecological important biopolymers and lipids
Source: PLoS One. 2018 Aug 30;13(8):e0202695. doi: 10.1371/journal.pone.0202695 (PMC6117010; doi:10.1371/journal.pone.0202695)
Supplement: S2 Fig — (PDF) [file pone.0202695.s002.pdf]

Plate-based screening of actively growing fungi

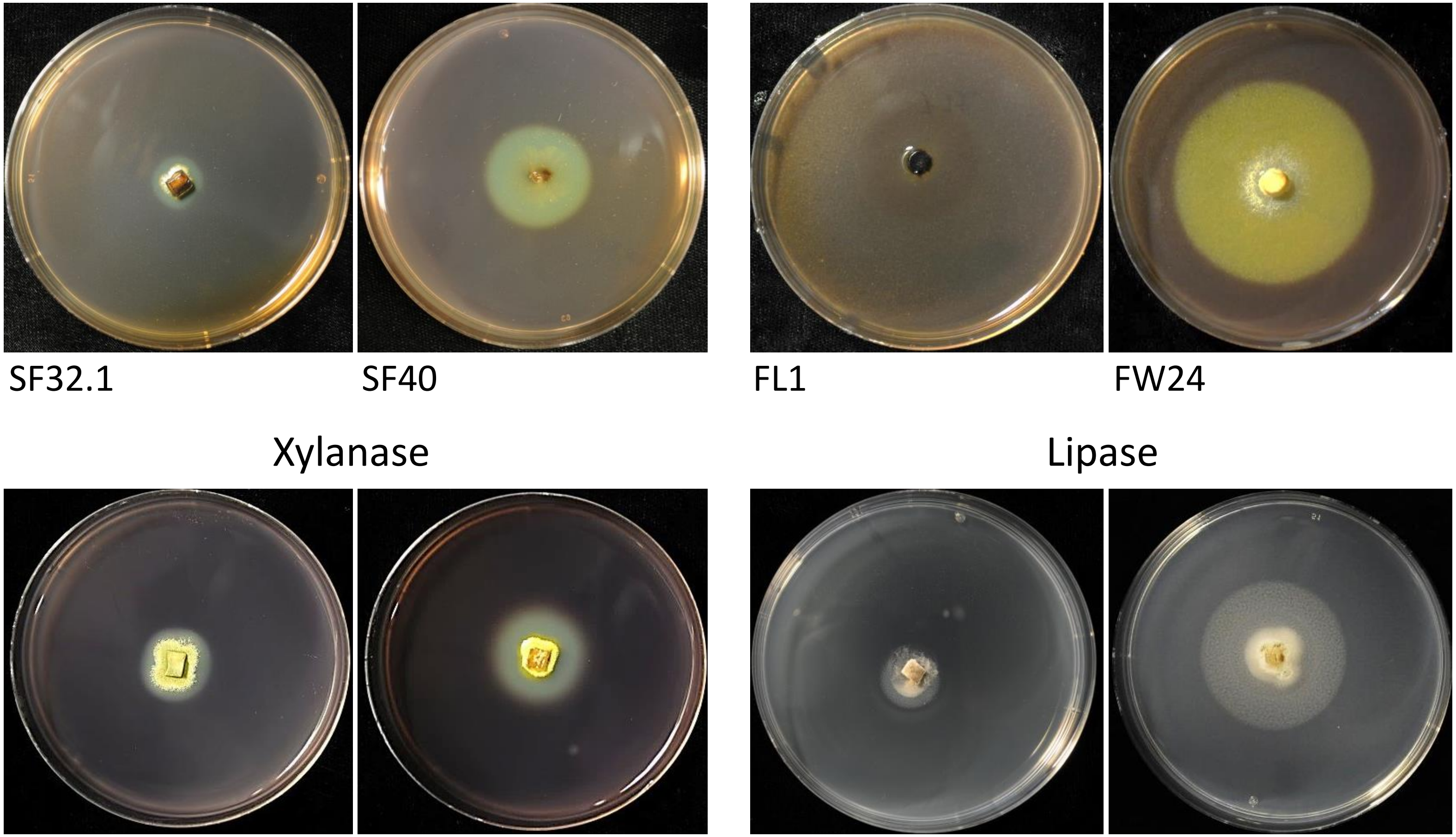

Cellulase: SF32.1, SF40

Chitinase: FL1, FW24

Xylanase: Fsh200, Fsh102

Lipase: SF2.2, Fsh102

Supporting Figure 2: Plate based enzyme activity assay. First picture in each group (Cellulase, Chitinase, Xylanase and Lipase) shows no or low specific activity. Second picture in each group shows high activity.
