# Supplementary material for: A unique fungal strain collection from Vietnam characterized for high performance degraders of bioecological important biopolymers and lipids
Source: PLoS One. 2018 Aug 30;13(8):e0202695. doi: 10.1371/journal.pone.0202695 (PMC6117010; doi:10.1371/journal.pone.0202695)
Supplement: S3 Fig — (PDF) [file pone.0202695.s003.pdf]

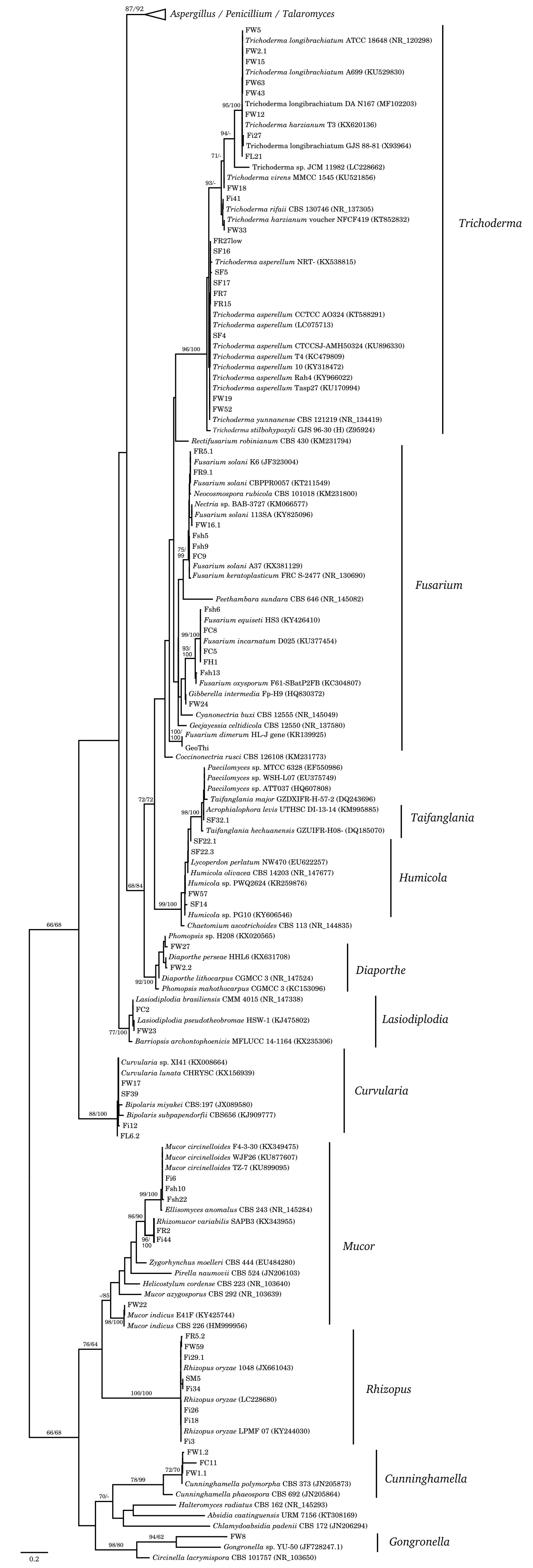
87/92
Aspergillus / Penicillium / Talaromyces
FW5
Trichoderma longibrachiatum ATCC 18648 (NR_120298)
FW2.1
FW15
Trichoderma longibrachiatum A699 (KU529830)
FW63
FW43
95/100
Trichoderma longibrachiatum DA N167 (MF102203)
FW12
Trichoderma harzianum T3 (KX620136)
94/-
Fi27
Trichoderma longibrachiatum GJS 88-81 (X93964)
71/-
FL21
Trichoderma sp. JCM 11982 (LC228662)
93/-
Trichoderma virens MMCC 1545 (KU521856)
FW18
Fi41
Trichoderma rifaii CBS 130746 (NR_137305)
Trichoderma harzianum voucher NFCF419 (KT852832)
FW33
Trichoderma
FR27low
SF16
Trichoderma asperellum NRT- (KX538815)
SF5
SF17
FR7
FR15
Trichoderma asperellum CCTCC AO324 (KT588291)
Trichoderma asperellum (LC075713)
SF4
96/100
Trichoderma asperellum CTCCSJ-AMH50324 (KU896330)
Trichoderma asperellum T4 (KC479809)
Trichoderma asperellum 10 (KY318472)
Trichoderma asperellum Rah4 (KY966022)
Trichoderma asperellum Tasp27 (KU170994)
FW19
FW52
Trichoderma yunnanense CBS 121219 (NR_134419)
Trichoderma stilbohypoxyli GJS 96-30 (H) (Z95924)
Rectifusarium robinianum CBS 430 (KM231794)
FR5.1
Fusarium solani K6 (JF323004)
FR9.1
Fusarium solani CBPPR0057 (KT211549)
Neocosmospora rubicola CBS 101018 (KM231800)
Nectria sp. BAB-3727 (KM066577)
Fusarium solani 113SA (KY825096)
FW16.1
Fsh5
75/
99
Fsh9
FC9
Fusarium solani A37 (KX381129)
Fusarium keratoplasticum FRC S-2477 (NR_130690)
Fusarium
Peethambara sundara CBS 646 (NR_145082)
Fsh6
Fusarium equiseti HS3 (KY426410)
99/100
FC8
93/
100
Fusarium incarnatum D025 (KU377454)
FC5
FH1
Fsh13
Fusarium oxysporum F61-SBatP2FB (KC304807)
Gibberella intermedia Fp-H9 (HQ830372)
FW24
Cyanonectria buxi CBS 12555 (NR_145049)
Geejayessia celtidicola CBS 12550 (NR_137580)
100/
100
Fusarium dimerum HL-J gene (KR139925)
GeoThi
Coccinonectria rusci CBS 126108 (KM231773)
Paecilomyces sp. MTCC 6328 (EF550986)
Paecilomyces sp. WSH-L07 (EU375749)
Paecilomyces sp. ATT037 (HQ607808)
72/72
Taifanglania major GZDXIFR-H-57-2 (DQ243696)
98/100
Acrophialophora levis UTHSC DI-13-14 (KM995885)
SF32.1
Taifanglania
Taifanglania hechuanensis GZUIFR-H08- (DQ185070)
SF22.1
SF22.3
Lycoperdon perlatum NW470 (EU622257)
Humicola olivacea CBS 14203 (NR_147677)
68/84
Humicola sp. PWQ2624 (KR259876)
Humicola
FW57
99/100
SF14
Humicola sp. PG10 (KY606546)
Chaetomium ascotrichoides CBS 113 (NR_144835)
66/68
Phomopsis sp. H208 (KX020565)
FW27
Diaporthe perseae HHL6 (KX631708)
FW2.2
Diaporthe
92/100
Diaporthe lithocarpus CGMCC 3 (NR_147524)
Phomopsis mahothocarpus CGMCC 3 (KC153096)
Lasiodiplodia brasiliensis CMM 4015 (NR_147338)
FC2
Lasiodiplodia pseudotheobromae HSW-1 (KJ475802)
Lasiodiplodia
FW23
77/100
Barriopsis archontophoenicis MFLUCC 14-1164 (KX235306)
Curvularia sp. XI41 (KX008664)
Curvularia lunata CHRYSC (KX156939)
FW17
Curvularia
SF39
Bipolaris miyakei CBS:197 (JX089580)
88/100
Bipolaris subpapendorfii CBS656 (KJ909777)
Fi12
FL6.2
Mucor circinelloides F4-3-30 (KX349475)
Mucor circinelloides WJF26 (KU877607)
Mucor circinelloides TZ-7 (KU899095)
Fi6
99/100
Fsh10
Fsh22
Ellisomyces anomalus CBS 243 (NR_145284)
86/90
Rhizomucor variabilis SAPB3 (KX343955)
FR2
96/
100
Fi44
Mucor
Zygorhynchus moelleri CBS 444 (EU484280)
Pirella naumovii CBS 524 (JN206103)
Helicostylum cordense CBS 223 (NR_103640)
-/85
Mucor azygosporus CBS 292 (NR_103639)
FW22
Mucor indicus E41F (KY425744)
98/100
Mucor indicus CBS 226 (HM999956)
FR5.2
76/64
FW59
Fi29.1
Rhizopus oryzae 1048 (JX661043)
SM5
Fi34
Rhizopus
100/100
Rhizopus oryzae (LC228680)
Fi26
Fi18
Rhizopus oryzae LPMF 07 (KY244030)
66/68
Fi3
FW1.2
FC11
72/70
FW1.1
78/99
Cunninghamella
Cunninghamella polymorpha CBS 373 (JN205873)
Cunninghamella phaeospora CBS 692 (JN205864)
70/-
Halteromyces radiatus CBS 162 (NR_145293)
Absidia caatinguensis URM 7156 (KT308169)
Chlamydoabsidia padenii CBS 172 (JN206294)
94/62
FW8
Gongronella
Gongronella sp. YU-50 (JF728247.1)
98/80
0.2
Circinella lacrymispora CBS 101757 (NR_103650)
