# Supplementary material for: A unique fungal strain collection from Vietnam characterized for high performance degraders of bioecological important biopolymers and lipids
Source: PLoS One. 2018 Aug 30;13(8):e0202695. doi: 10.1371/journal.pone.0202695 (PMC6117010; doi:10.1371/journal.pone.0202695)
Supplement: S4 Fig — (PDF) [file pone.0202695.s004.pdf]

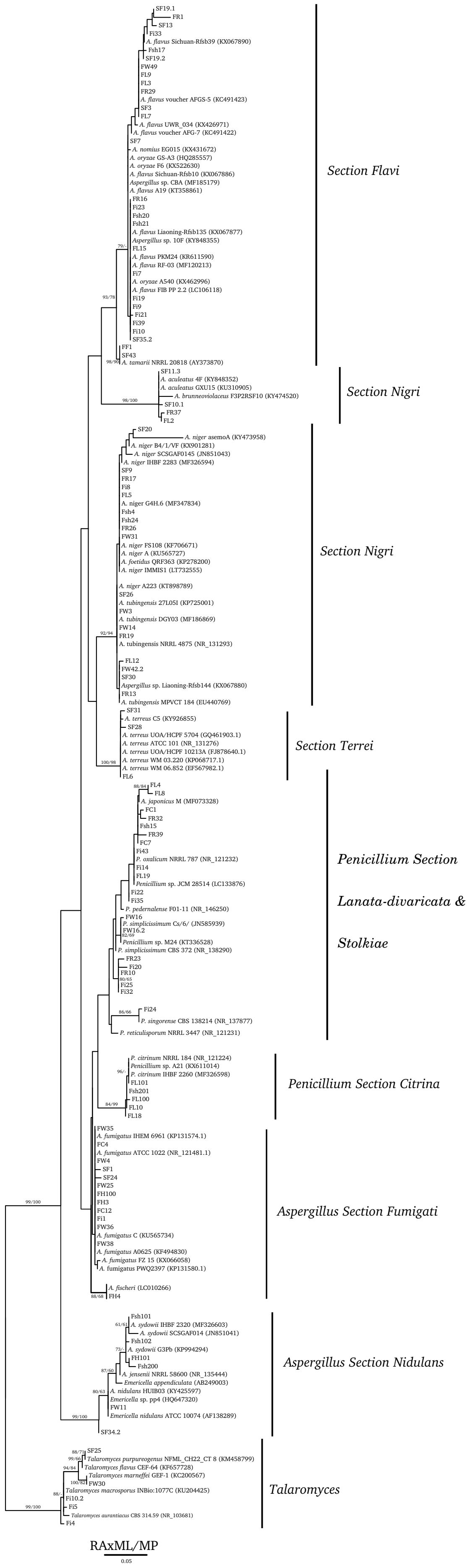
SF19.1
FR1
SF13
Fi33
A. flavus Sichuan-Rfsb39 (KX067890)
Fsh17
SF19.2
FW49
FL9
FL3
FR29
A. flavus voucher AFGS-5 (KC491423)
SF3
FL7
A. flavus UWR_034 (KX426971)
A. flavus voucher AFG-7 (KC491422)
SF7
A. nomius EG015 (KX431672)
A. oryzae GS-A3 (HQ285557)
A. oryzae F6 (KX522630)
A. flavus Sichuan-Rfsb10 (KX067886)
Aspergillus sp. CBA (MF185179)
A. flavus A19 (KT358861)
FR16
Fi23
Fsh20
Fsh21
A. flavus Liaoning-Rfsb135 (KX067877)
Aspergillus sp. 10F (KY848355)
79/-
FL15
A. flavus PKM24 (KR611590)
A. flavus RF-03 (MF120213)
Fi7
A. oryzae A540 (KX462996)
A. flavus FIB PP 2.2 (LC106118)
93/78
Fi19
Fi9
Fi21
Fi39
Fi10
SF35.2
FF1
SF43
98/90
A. tamarii NRRL 20818 (AY373870)
Section Flavi
SF11.3
A. aculeatus 4F (KY848352)
A. aculeatus GXU15 (KU310905)
A. brunneoviolaceus F3P2RSF10 (KY474520)
98/100
SF10.1
FR37
FL2
Section Nigri
SF20
A. niger asemoA (KY473958)
A. niger B4/1/VF (KX901281)
A. niger SCSGAF0145 (JN851043)
A. niger IHBF 2283 (MF326594)
SF9
FR17
Fi8
FL5
A. niger G4H.6 (MF347834)
Fsh4
Fsh24
FR26
FW31
A. niger FS108 (KF706671)
A. niger A (KU565727)
A. foetidus QRF363 (KP278200)
A. niger IMMIS1 (LT732555)
A. niger A223 (KT898789)
SF26
A. tubingensis 27L0SI (KP725001)
FW3
A. tubingensis DGY03 (MF186869)
FW14
92/94
FR19
A. tubingensis NRRL 4875 (NR_131293)
FL12
FW42.2
SF30
Aspergillus sp. Liaoning-Rfsb144 (KX067880)
FR13
A. tubingensis MPVCT 184 (EU440769)
Section Nigri
SF31
A. terreus C5 (KY926855)
SF28
A. terreus UOA/HCPF 5704 (GQ461903.1)
A. terreus ATCC 101 (NR_131276)
A. terreus UOA/HCPF 10213A (FJ878640.1)
A. terreus WM 03.220 (KP068717.1)
100/98
A. terreus WM 06.852 (EF567982.1)
FL6
Section Terrei
88/84
FL4
FL8
A. japonicus M (MF073328)
FC1
FR32
Fsh15
FR39
FC7
Fi43
P. oxalicum NRRL 787 (NR_121232)
Fi14
FL19
Penicillium sp. JCM 28514 (LC133876)
Fi22
Fi35
P. pedernalense F01-11 (NR_146250)
FW16
P. simplicissimum Cs/6/ (JN585939)
FW16.2
82/69
Penicillium sp. M24 (KT336528)
P. simplicissimum CBS 372 (NR_138290)
FR23
Fi20
FR10
80/65
Fi25
Fi32
86/66
Fi24
P. singorense CBS 138214 (NR_137877)
P. reticulisporum NRRL 3447 (NR_121231)
Penicillium Section
Lanata-divaricata &
Stolkiae
P. citrinum NRRL 184 (NR_121224)
Penicillium sp. A21 (KX611014)
96/-
P. citrinum IHBF 2260 (MF326598)
FL101
Fsh201
FL100
84/99
FL10
FL18
Penicillium Section Citrina
FW35
A. fumigatus IHEM 6961 (KP131574.1)
FC4
A. fumigatus ATCC 1022 (NR_121481.1)
FW4
SF1
SF24
FW25
FH100
FH3
FC12
Fi1
FW36
A. fumigatus C (KU565734)
FW38
A. fumigatus A0625 (KF494830)
A. fumigatus FZ 15 (KX066058)
A. fumigatus PWQ2397 (KP131580.1)
A. fischeri (LC010266)
88/68
FH4
Aspergillus Section Fumigati
99/100
Fsh101
61/91
A. sydowii IHBF 2320 (MF326603)
A. sydowii SCSGAF014 (JN851041)
Fsh102
73/-
A. sydowii G3Pb (KP994294)
FH101
Fsh200
87/60
A. jensenii NRRL 58600 (NR_135444)
Emericella appendiculata (AB249003)
80/63
A. nidulans HUIB03 (KY425597)
Emericella sp. pp4 (HQ647320)
FW11
99/100
Emericella nidulans ATCC 10074 (AF138289)
SF34.2
Aspergillus Section Nidulans
88/71
SF25
99/66
Talaromyces purpureogenus NFML_CH22_CT 8 (KM458799)
94/84
Talaromyces flavus CEF-64 (KF657728)
Talaromyces marneffei GEF-1 (KC200567)
100/82
FW30
Talaromyces macrosporus INBio:1077C (KU204425)
88/-
Fi10.2
Fi5
99/100
Talaromyces aurantiacus CBS 314.59 (NR_103681)
Fi4
Talaromyces
RAxML/MP
0.05
